# The safe development paradox of the United States regulatory floodplain

**DOI:** 10.1371/journal.pone.0311718

**Published:** 2024-12-31

**Authors:** Georgina M. Sanchez, Margaret A. Lawrimore, Anna Petrasova, John B. Vogler, Elyssa L. Collins, Vaclav Petras, Truffaut Harper, Emma J. Butzler, Ross K. Meentemeyer

**Affiliations:** 1 Center for Geospatial Analytics, North Carolina State University, Raleigh, North Carolina, United States of America; 2 Department of Forestry and Environmental Resources, North Carolina State University, Raleigh, North Carolina, United States of America; Yeungnam University, REPUBLIC OF KOREA

## Abstract

In the United States, requirements for flood insurance, development restrictions, and federal buyout program eligibility rely on regulatory designation of hazardous zones, i.e., inside or outside the 100-year floodplain. Extensive research has investigated floodplain development patterns across different geographies, times, and scales, yet the impacts, and potential unintended consequences, of floodplain policies beyond their boundaries have not been empirically examined. We posit that the regulatory 100-year floodplain presents a “safe development paradox”, whereby attempts to reduce flood risk paradoxically intensifies it by promoting development in and near flood-prone areas. We conducted the first comprehensive national assessment of historical and future development patterns related to the regulatory 100-year floodplain, examining the spatial distribution of developed land within increasingly distant 250-m zones from floodplain boundaries. We found a disproportionate concentration of developed land (24% or 89,080 km^2^ of developed land by 2019) in zones immediately adjacent to the floodplain, a trend observed at the national, state, and county levels. Nationwide projections suggest that approximately 22% of all anticipated growth from 2020 to 2060 is likely to occur within 250 m from the 100-year floodplain, equivalent to 6,900 km^2^ of new development (SD = 2,842 km^2^). Understanding and anticipating the influence of flood management policies on current and future land use decisions is crucial for effective planning and mitigation strategies.

## 1. Introduction

Floods present substantial risks to life and property, with economic damages costing the United States $8.2 billion annually [1]. There is consequently increasing pressure on governments to enact policies that reduce flood damage. Paradoxically, many government policies and flood management strategies intended to mitigate flood risks unintentionally contribute to increased flood exposure by creating false senses of security and therefore driving development in flood-prone areas [2–5]. This phenomenon, known as the "safe development paradox," has been observed in various studies [6, 7].

Research on the safe development paradox (SDP) has predominantly focused on the "levee effect," that is, the perception that flood protection infrastructure, such as levees, entirely removes the threat of flooding. This misperception leads to increased development in flood-prone areas, raising the potential for greater damage if the levee fails during a flood event that exceeds its design [8–12].”Policy effects,” in contrast, are understudied, and understanding their role in shaping land use decisions is crucial to effectively plan and adapt to the escalating threats posed by flooding.

One highly influential policy with potential SDP implications is the U.S. Federal Emergency Management Agency (FEMA) designation of high-risk flood zones, commonly known as floodplain mapping. The primary objective of floodplain maps is to minimize the adverse impact of floods by identifying areas of risk, encouraging floodplain management, providing guidance for land use decisions, and fostering the adoption of flood-resilient development practices [13, 14]. However, FEMA’s National Flood Insurance Program (NFIP), which provides federal funding for flood damage and relies in part on FEMA flood maps [15], may unintentionally incentivize development in flood-prone areas [16–20]. Currently, 13.3% of the U.S. population resides in areas designated by FEMA as high-risk [1], that is, within a 100-year floodplain where there is a 1% chance of flooding in any given year, or a 26% chance of flooding over a 30-year mortgage. Recent trends indicate that an increasing proportion of the total population in the conterminous United States (CONUS) will reside within a floodplain in the future (from 13.3% to 15.6–15.8% by 2050 [1]). Moreover, in the U.S., the effects of climate change on sea-level rise and extreme weather events are projected to expand areas of increased flood risk by up to 45% by the end of the century [21].

Numerous studies highlight the limitations of current floodplain maps in accurately representing flood risk [1, 22, 33]. These maps often fail to capture the dynamic nature of flood hazards, resulting in the underestimation of risk and creating misleading perceptions of safety [23]. Infrastructure located just a few meters outside the designated floodplain are not subject to flood regulatory measures, yet floods can and do breach floodplain boundaries unexpectedly [24]. As a result, homeowners and businesses in areas immediately outside the floodplain may remain unaware of their risks and thus unprepared for floods. The limitations and static nature of floodplain maps [1, 22, 23, 25], therefore, hamper effective floodplain management and increase vulnerability to flood-related damages. Despite these limitations, many prior studies assessing current and future flood exposure constrained their study extent to within high-risk flood zones (e.g., 100-year floodplain [1, 21, 26–29]). Surprisingly, little research has investigated the influence of floodplain maps on development patterns outside floodplain boundaries [2, 30, 31]. This knowledge gap significantly challenges our understanding of the broader implications of floodplain management policies and potential unintended consequences. It also underscores the need for more comprehensive studies that conceptualize floodplain mapping as a case of the SDP and investigate its influence on development patterns, both within and beyond the designated flood zones.

Our study aims to conduct a comprehensive assessment of the influence of the 100-year floodplain designation on development patterns across the CONUS and to explore the potential implications for future flood exposure. We address the following research question: Does FEMA’s regulatory 100-year floodplain present a safe development paradox? To answer this question, our objectives are twofold: First, we examined how historical (2001 to 2019) and future (2060) development patterns vary with proximity to the 100-year floodplain and across geographic scales. Second, to capture the spatial heterogeneity of urban growth, we analyzed the extent and distribution of development inside the floodplain, within eight 250-m concentric distance zones from the edge of floodplain boundaries, and in areas greater than 2 km from floodplain boundaries. Summarized at the county, state, and national levels, our findings quantify and highlight an ever-increasing buildup of exposed infrastructure in areas immediately adjacent to floodplains, where flood risk is becoming increasingly uncertain under a changing climate. Local and regional decision makers can use these findings, for example, improving awareness of at-risk communities to the limitations of existing flood zone delineations, justifying updates to flood hazard maps, supporting reforms to planning and zoning practices, and prioritizing mitigation strategies in these areas of emerging flood risk. Our examination of the relationship between FEMA’s 100-year floodplain and changing development patterns contributes to a broader understanding of how land use decisions are influenced by non-structural flood management strategies. This knowledge is crucial for effective policy making, planning, and implementation of adaptation measures to mitigate flood risks and protect communities from potential flood damage.

## 2. Methods

### 2.1. Study system

**Fig 1:**
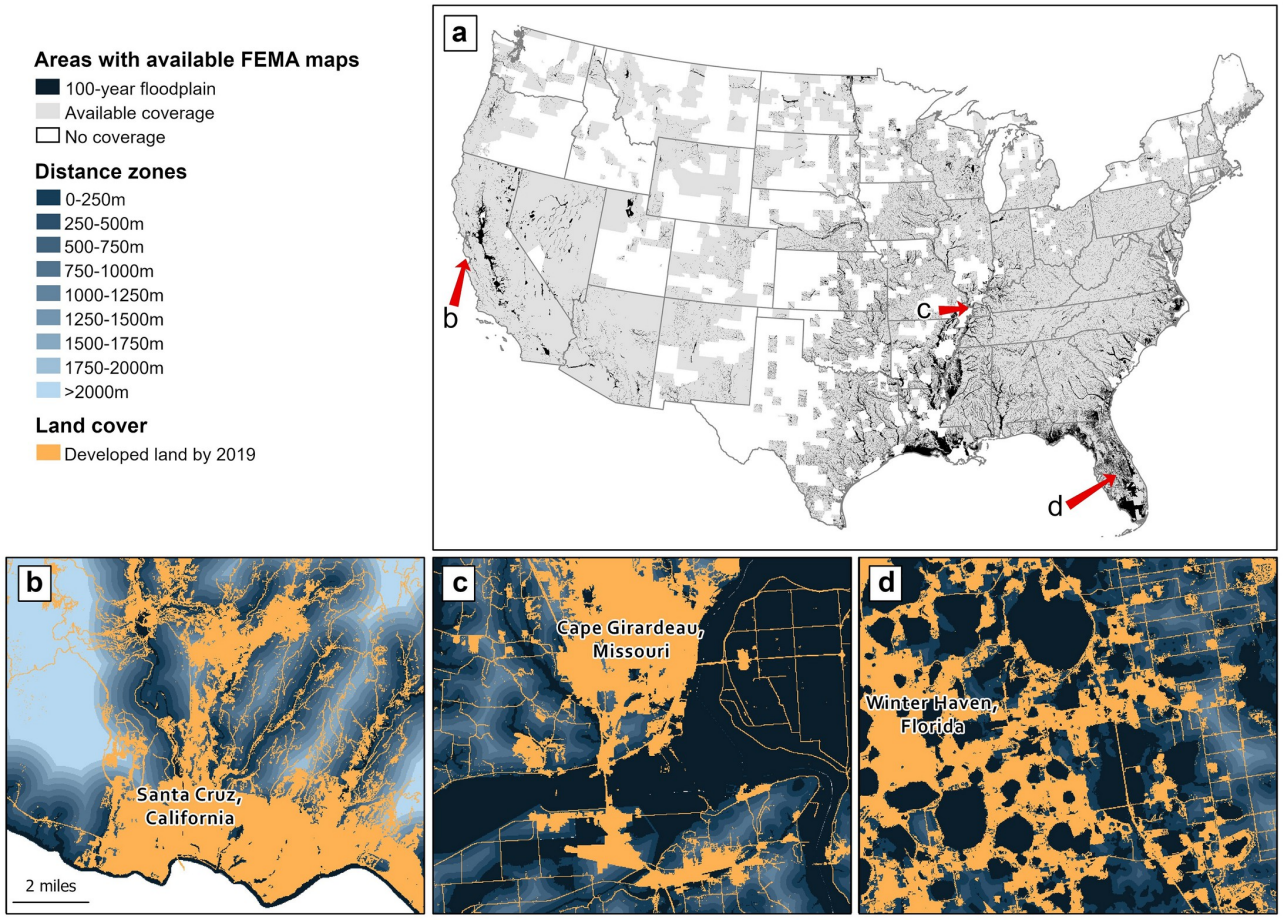
(a) The study system comprises 2,330 counties across the conterminous U.S with complete coverage of the Federal Emergency Management Agency’s (FEMA) 100-year floodplain maps. Developed land cover, the 100-year floodplain, and distance zones outside the floodplain shown for the communities of (b) Santa Cruz, CA, (c) Cape Girardeau, MO, and (d) Winter Haven, FL. State boundaries are public domain data sourced from the U.S. Census Bureau’s TIGER/Line Shapefiles. Floodplain coverage is public domain data sourced from the U.S. Federal Emergency Management Agency’s Flood Insurance Rate Maps. Land cover is public domain data sourced from the U.S. Geological Survey’s National Land Cover Database. All other data produced by the authors.

To assess the effect of federally designated high-risk flood zones on the distribution of observed and projected development patterns, we considered the 2,330 counties (75% of the 3,108 counties in the CONUS) in the CONUS with available FEMA Flood Insurance Rate Maps (FIRM), collected as of 2021, that define the 100-year floodplain extent [14] (Fig 1a). Of the 3,108 total counties in the CONUS, we excluded from our analysis 715 (23%) unmapped counties and 63 (2%) partially mapped counties with incomplete delineation of the 100-year floodplain. As of 2023, FEMA has no national regularized schedule for surveying and mapping flood hazards (i.e., National Flood Hazard Layer [NFHL] maps); as a result, only 63% of the U.S. land area is mapped [23, 32]. Given the high cost of creating and updating maps—ranging from $5,000 to $10,000 per linear mile of stream—and the tendency to prioritize highly populated and frequently flooded areas, gaps are common in rural communities and small catchments [21, 23, 32]. Currently, about 90% of the U.S. population lives in a community with available coverage [23]. Though we recognize the availability of other spatially complete 100-year floodplain maps (e.g., [32]), we intentionally focused this analysis on the primary federal source of flood regulatory information used by local governments to guide planning decisions.

### 2.2. Data sources

**Fig 2:**
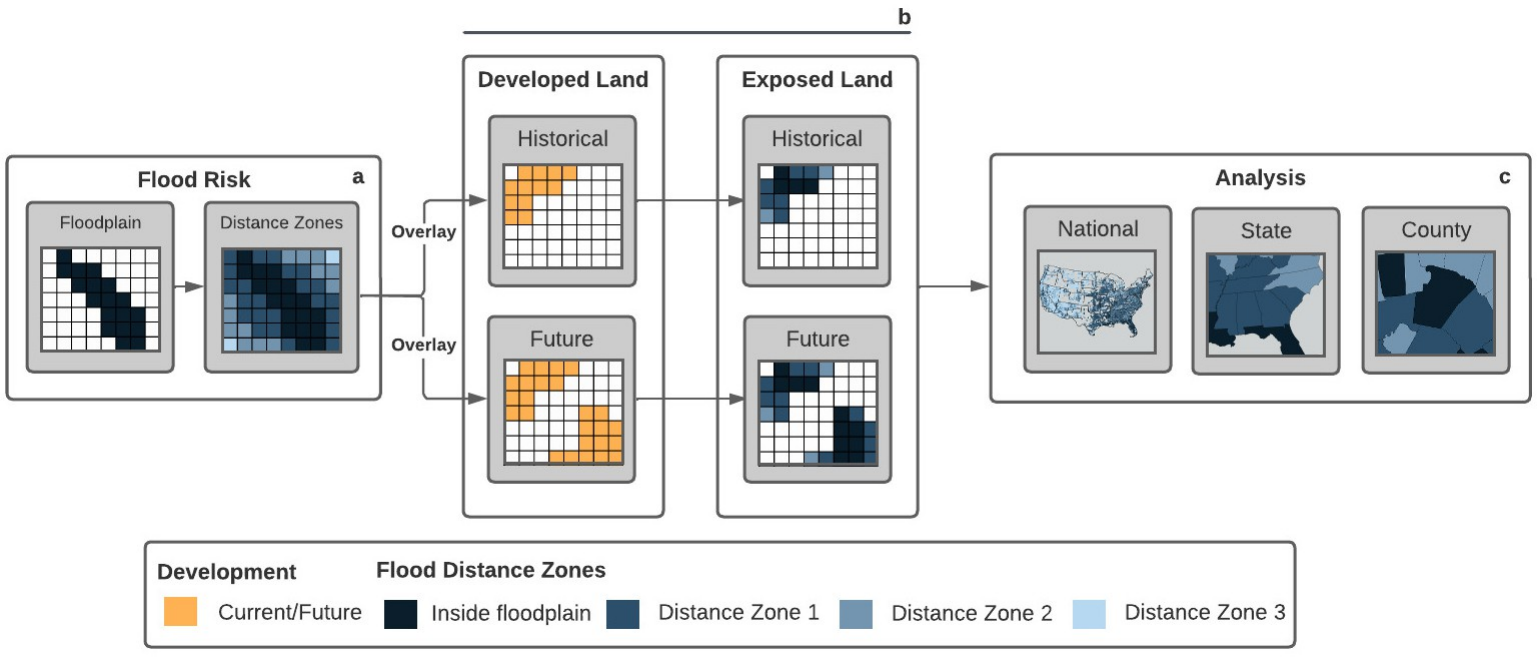
Workflow to quantify the extent (km^2^) and spatial distribution of developed land relative to high-risk flood areas and across multiple scales. (a) Available 100-year floodplain maps used to classify land into ten distance zones within and outside the floodplain: inside floodplain, 0–250 m, 250–500 m, 500–750 m, 750–1000 m, 1000–1250 m, 1250–1500 m, 1500–1750 m, 1750–2000 m, and >2000 m from floodplain edge. (b) Historical (2001, 2019) and future (2060) developed land overlaid with distance zones to quantify amounts (km^2^) of development per distance zone, the proportion of developed land area per zone, and changes in developed land area over evaluated periods (2001–2019, 2019–2060). (c) Results analyzed at the national, state, and county levels.

We analyzed the 2,330 counties within the CONUS with available 100-year floodplains (also referred to as mapped floodplains) as designated by the FEMA NFHL [14]. Mapped floodplains are available in vector format and are publicly accessible at FEMA’s Map Service Center (https://msc.fema.gov/portal/home). The flood designations included in FEMA FIRM zones are “significant” (i.e., high or >1% annual chance of flooding), “minimal” (i.e., <1% annual chance of flooding), and “undetermined” (i.e., unmapped areas) flood risk. We converted the floodplain layer into a 30-m resolution gridded raster, aligning with the resolution of the National Land Cover Database used in the next step, and reclassified designations into two categories, inside the 100-year floodplain and outside of the 100-year floodplain (Fig 2a). Unmapped or undetermined cells are excluded from the study.

We used the National Land Cover Database (NLCD) to identify locations of developed land cover [33] for the years 2001 and 2019 in all counties with available floodplain maps. The NLCD land cover data are 30-m resolution gridded rasters with eight general land cover classes (i.e., developed, water, barren, forest, shrubland, herbaceous, planted/cultivated, and wetlands) and 16 subclasses. For our analysis, we reclassified these land cover classes into two categories: “developed” land (Fig 2b) includes open space developed and low-, medium-, and high-intensity developed; “undeveloped” includes all other classes.

### 2.3. Simulating future development

We used the FUTure Urban-Regional Environment Simulation (FUTURES version 3.0 [34]) model to project urban growth through the year 2060 across the CONUS. FUTURES is a land change model that simulates spatially interactive patterns and processes of development in response to scenarios defined by the user. Spatially interactive processes take into account how conditions in one location, whether observed or simulated, influence conditions in another location. For example, when new urban growth occurs in one area, it can impact the likelihood of growth in the surrounding areas. FUTURES simulates development patterns and their interactions by integrating four submodels that consider site suitability for land change (POTENTIAL), per capita land consumption in a region (DEMAND), the spatial structure of urbanization, and adaptive response to changing environmental conditions (CLIMATE FORCINGS). To parameterize simulations, we followed [34–37] using historical (2001, 2004, 2006, 2008, 2011, 2013, 2016, 2019) developed land cover [33], a suite of predictor variables, and estimates of historical and projected population [38]. Section 1 provides details about predictor variables and model coefficients used in this study (S1 File). To validate the accuracy of land change patterns simulated by the FUTURES model, we used a hindcast approach whereby we evaluated the model’s accuracy to predict historical conditions of land change. Model performance is comparable to previous implementations of the FUTURES model following historical trajectories of growth [34–37, 39] and other modeling frameworks [40–42]. Supplementary Information provides details about model validation metrics (S1–S5 Figs), including the spatial distribution of simulation accuracy assessed through the figure of merit, mean allocation error, and mean quantity error across the study region (S2–S4 Figs).

The FUTURES model has consistently demonstrated accuracy in capturing new development patterns [34–37, 39] and is cited in over 100 landscape dynamics studies, supporting its use in our analysis. We used FUTURES open source GRASS GIS add-on [43] to compute simulations of urban growth from the year 2020 through 2060 in annual timesteps. Urban planning, flood policy, and mitigation often consider long-term horizons to address future needs and challenges. Our 40-year simulation period is long enough to reveal regional urbanization trends, yet not so distant that it would introduce multiple demographic turning points [44] that could shift projections from our selected “business as usual” growth scenario. This approach considers the potential effects of current flood risk regulations on future development, assuming no human adaptation in response to increasing future flood hazards [34] or alterations to existing policies intended to mitigate flood damage beyond high-risk flood zones [25]. At the time of our analysis, no federally recognized mapping effort comprehensively considered the impact of climate change on the spatial distribution of future flood risk. Therefore, we excluded consideration of climate change impacts from future simulations, and this application of FUTURES 3.0 does not incorporate the CLIMATE FORCINGS submodel. Although economic and demographic projections indicate significant demand for new development across the nation in the coming decades [38], long-term projections carry inherent uncertainties regarding the timing and location of new growth. To address uncertainty associated with stochastic variability in the evaluated scenario, we conducted 20 stochastic iterations, generating probability maps of areas likely to experience new development under the business-as-usual growth scenario.

### 2.4. Comprehensive national assessment

To quantify the extent and distribution of developed land change within and beyond the 100-year floodplain, we analyzed historical (2001, 2019) and future (2060) developed land cover within ten zones. In addition to an “inside floodplain” zone, we generated eight concentric 250-m wide zones (0–250 m, 250–500 m, 500–750 m, 750–1000 m, 1000–1250 m, 1250–1500 m, 1500–1750 m, 1750–2000 m) that extend from the floodplain boundary to 2 km outside the floodplain, and a tenth zone that includes areas at 2 km and beyond the floodplain boundary (all distances are Euclidean). We selected these zones in an effort to balance high-resolution results with the computational expense of analysis over large geographic extents (CONUS). Additionally, we considered zone-size suitability for floodplain management and planning applications. The choice of a 250-m-wide buffer is based on the approximate distance of 1–2 mid-size U.S. city blocks. This buffer size is small enough to captures intra-city variations while being large enough to efficiently track urban expansion over large areas. Furthermore, aggregating developed land located more than 2 km from the floodplain boundary into one distance zone allowed us to analyze and contrast development in adjacent floodplain surroundings with more distant development.

We summed developed land area (historical, current, and future) across the CONUS and within each state and county (Fig 2c) and calculated the percent of these development totals that occur in each distance zone. We then calculated, for each distance zone, the total developed land area in 2001, 2019, and 2060 and the percent change in developed land area over time (2001–2019, 2019–2060). To account for disproportionately larger zone sizes (e.g., generated by buffering sinuous rivers across the nation’s large stream network), we calculated the percent of the total land area in each zone that is developed land. This approach effectively normalizes developed land areas by the corresponding distance zone’s total land area and aids comparison. We excluded open water bodies (as classified by NLCD) and permanently protected lands [45] from each zone’s total land area. We analyzed results at the national, state, and county levels (Fig 2c) by zone, time step, and time period for all 2,330 counties considered in the study. At the county level, we tested for significant differences (p < 0.05 two-sample unequal variance t-tests) in mean amounts of developed land between distance zones and over time (2001–2019, 2019–2060) using two-sample unequal variance t-tests.

## 3. Results

### 3.1. National trends and projections

**Fig 3:**
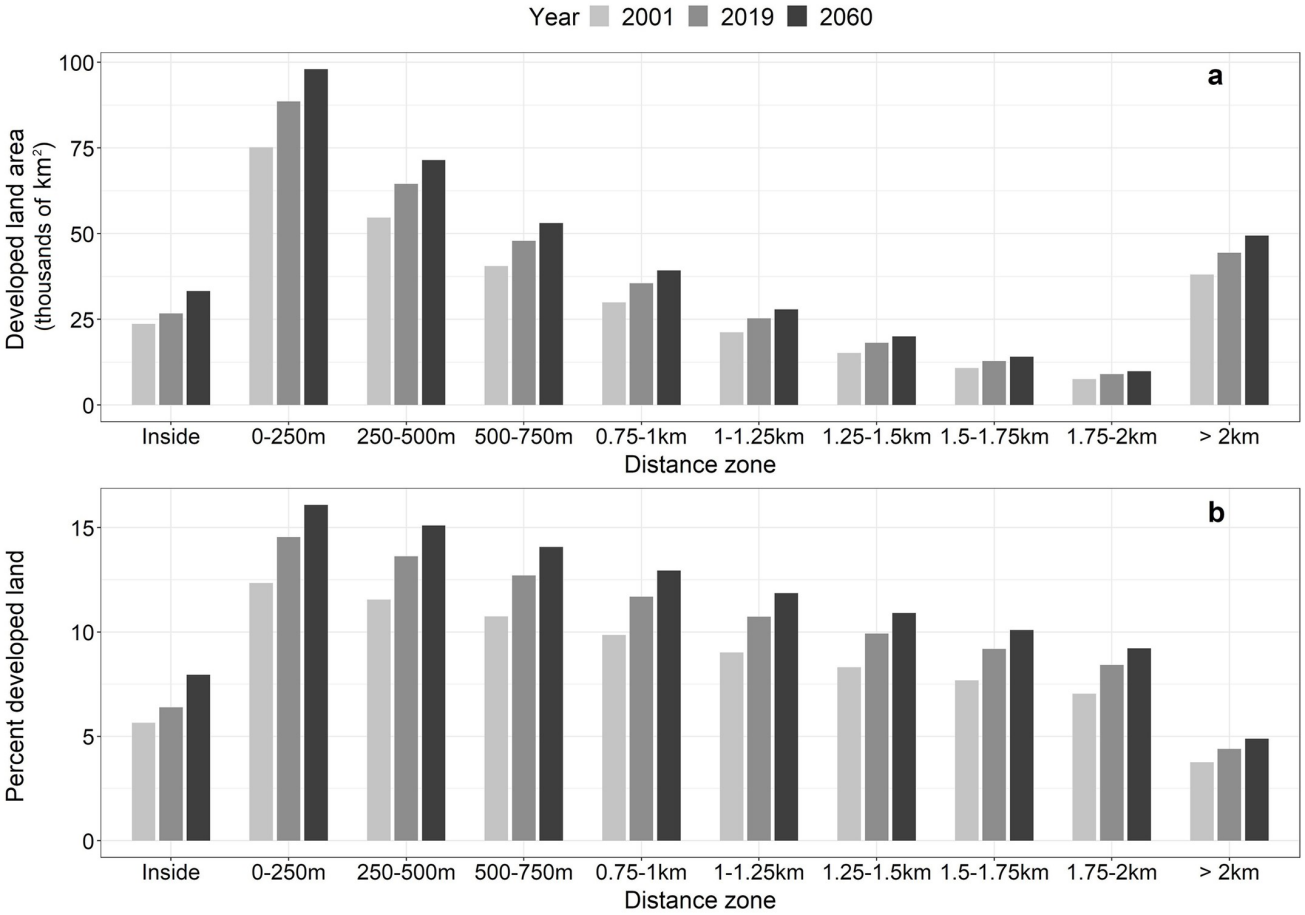
Distribution of developed land over time and with proximity to the 100-year floodplain boundary for the contiguous U.S. (CONUS). (a) Total amounts of developed land area (thousands of km^2^) and (b) percent developed land (i.e., as a proportion of total land area in each zone) shown for each distance zone and year (2001, 2019, 2060) for the CONUS. Values calculated from the 2,330 U.S. counties included in this study. Total land areas exclude open water bodies and permanently protected areas.

At the national level, we found that the total area of developed land (Fig 3a) and the proportion of developed land (Fig 3b) in each 250 m distance zone increases with proximity to the floodplain (Fig 3). For example, in 2019, we identified 89,080 km^2^ of total developed land in the 0–250 m zone adjacent to floodplains (Fig 3a and Section 2 in S1 File). This amount not only represents 24% of all developed land in the study system but also the highest percentage of developed land relative to zone area (15%) for any of the distance zones in 2019 (Fig 3b). The distribution of percent of developed land by zone increases from 4% in areas beyond 2 km of the floodplain to 14% in the 250–500 m distance zone. By comparison, 6% of land inside the floodplain was developed in 2019. We found similar trends of increasing total developed land area and increasing percent developed land with proximity to the floodplain in both the 2001 data and the 2060 projections (Fig 3 and Section 2 in S1 File).

Our analysis of developed land change (2001–2019) revealed that the highest increase in developed land area (13,460 km^2^) occurred in the 0–250 m zone, an 18% increase from 2001. Our urban growth simulations projected an additional 6,900 km^2^ (SD = 2,842 km^2^) of development in this zone by 2060, increasing 8% from 2019. Additionally, the 0–250 m zone is projected to have the largest total area of projected development of all the distance zones (Section 2 in S1 File). Percent change in developed land area between 2001 and 2019 was relatively consistent across all distance zones outside of the floodplain, ranging from 17–20%. Similarly, percent change in projected development (2019–2060) in distance zones outside of the floodplain remained consistent at 7–8% (Section 2 in S1 File). By contrast, total developed land area within the floodplain increased by 13% (3,123 km^2^) between 2001 and 2019, and our simulations projected an 18% increase (4,840 km^2^) in developed land area between 2019 and 2060 (Fig 3a and Section 1 in S1 File).

### 3.2. State trends and projections

**Fig 4:**
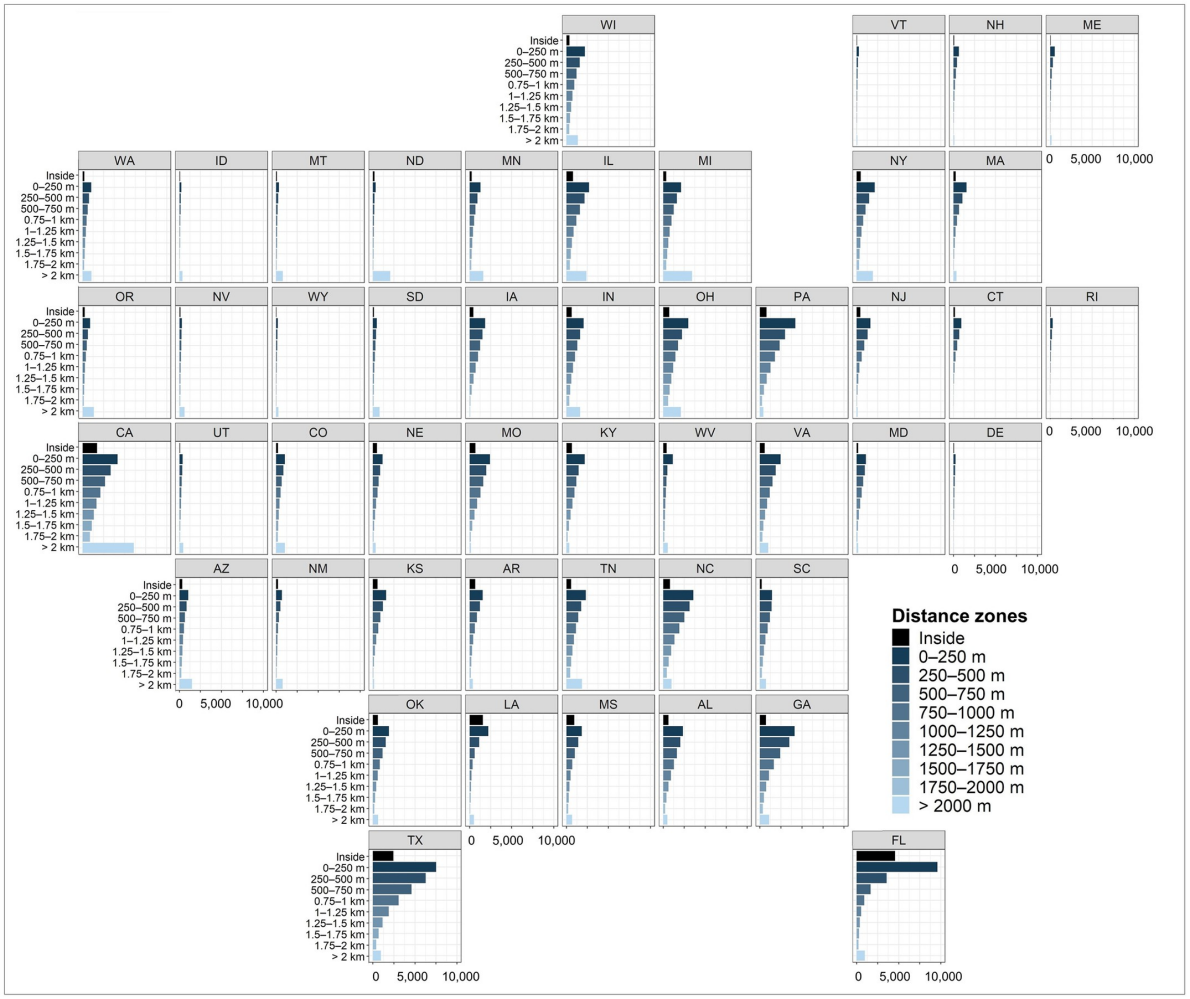
Distribution of total developed land area (km^2^) in 2019 summarized by distance zone and state. Two-letter state abbreviations used.

**Fig 5:**
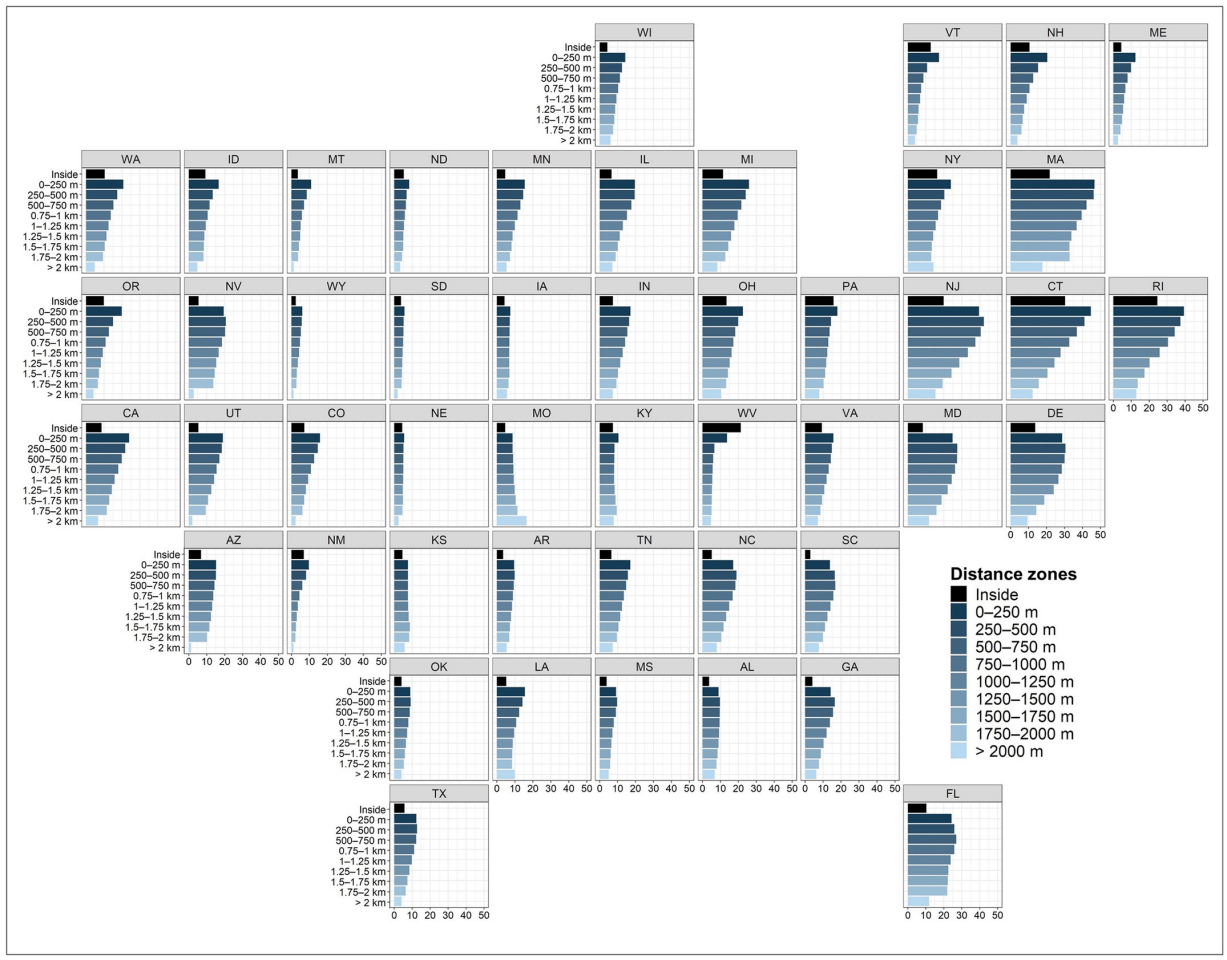
Distribution of percent developed land (i.e., proportion of total land area in each zone) in 2019 summarized by distance zone and state. Total land areas excludes open water bodies and permanently protected areas. Two-letter state abbreviations used.

We found development patterns at the state level to be consistent with national trends in several ways. First, in 2019, developed land area (km^2^) within 2 km of the 100-year floodplain increased with proximity to the floodplain in every state, with the highest amounts of development occurring in the 0–250 m zone adjacent to floodplains (Fig 4). In Florida, nearly 9,600 km^2^ of developed land in 2019 was concentrated in the 0–250 m zone, the highest of any state, accounting for 24% of the zone’s land area (Figs 4 and 5). Texas had approximately 7,500 km^2^ of development in this zone (12% of the zone’s land area), putting it in second place, followed by Georgia (14%), Pennsylvania (18%), and California (24%), each with nearly 4,200 km^2^ of developed land in the 0–250 m zone (Figs 4 and 5). Second, in a majority of states (63%), the percentage of developed land in each 250 m zone increased with proximity to the floodplain in 2019, peaking in the 0–250 m zone (Fig 5). Across all states, the percent developed land, on average, is 18% in the 0–250 m zone, 9% inside the floodplain, and 7–16% in remaining distance zones (Fig 5). The percent developed land in the 0–250 m zone exceeded 30% in several Northeastern states (Delaware, Connecticut, Massachusetts, Rhode Island, New Jersey; Fig 5). Third, in nearly every state, we observed a sharp decline in both the amount (Fig 4) and percent (Fig 5) of developed land inside the floodplain when compared to the 0–250 m zone. West Virginia is a notable exception, being the only state where the highest percentage of developed land (21%) is located in the floodplain (Fig 5). Florida had the largest area of development (4,562 km^2^) inside the floodplain of any state (Fig 4), representing 20% of the state’s total developed land area. The proportion of each state’s total developed land area located in the 0–250 m zone ranged from 9% in North Dakota (326 km^2^) to 42% in Florida (9,599 km^2^). Sixteen states (Arkansas, Connecticut, Florida, Georgia, Kansas, Kentucky, Louisiana, Maine, Massachusetts, New Hampshire, New Jersey, Pennsylvania, Rhode Island, Texas, Vermont, and West Virginia) have more than 25% of their overall developed land area located within the 0–250 m zone (Fig 4 and S1 Table).

**Fig 6:**
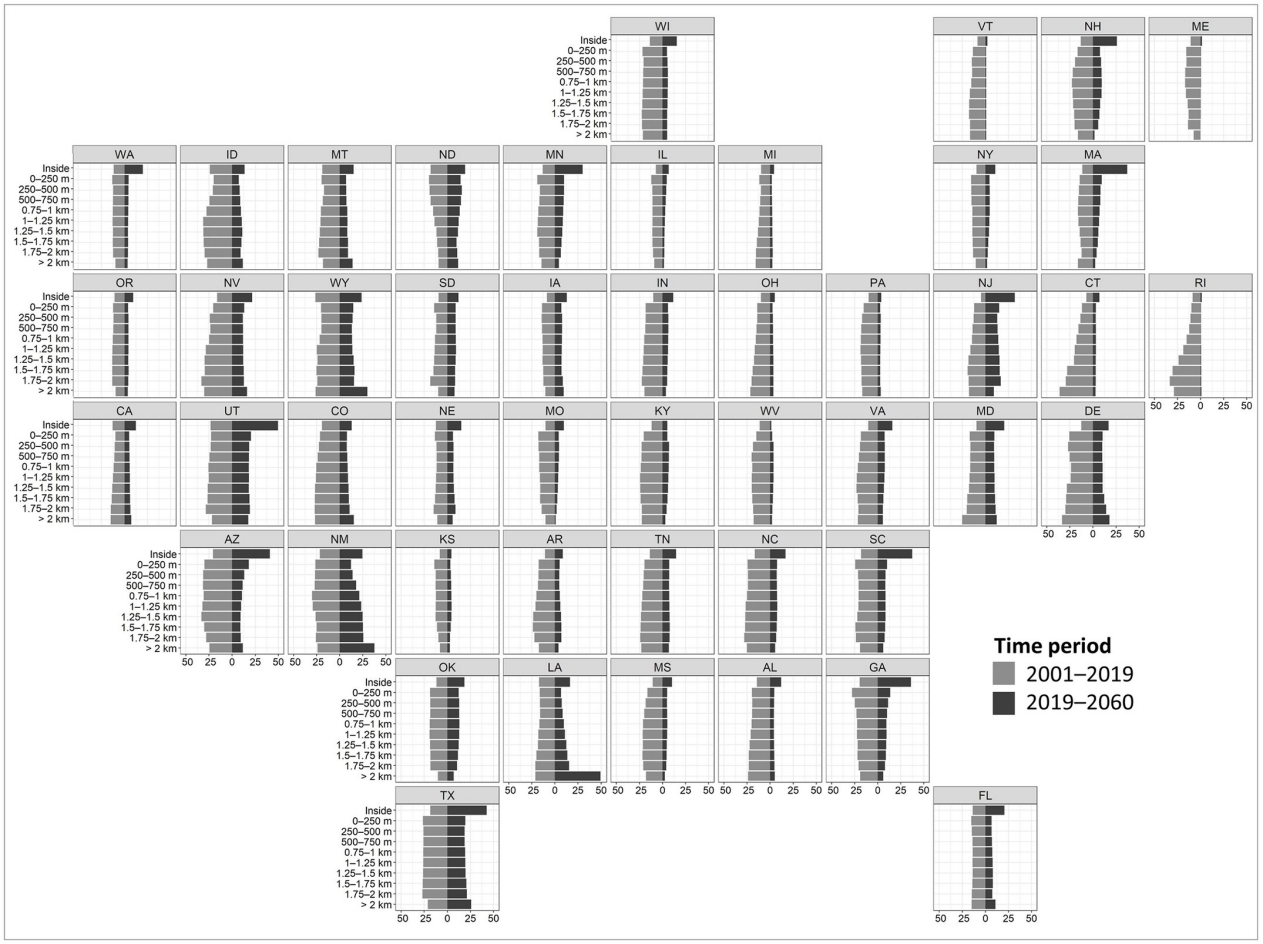
Percent change in developed land area in each distance zone over the 2001–2019 and 2019–2060 time periods.

Percent change in developed land area varied by region and time period (Fig 6). During the historical period (2001–2019), states in the Pacific West experienced consistent growth in developed land across all distance zones, with California, Oregon, and Washington ranging from 9% to 15% in percent change (Fig 6). In contrast, the Mountain West states showed greater variability in percent change during the same period. While North Dakota, Arizona, and New Mexico displayed greater percent change with proximity to the floodplain, the great majority of states (Montana, Idaho, Wyoming, Utah, Colorado, Nebraska, Nevada, South Dakota) displayed the opposite trend. In the Midwest, percent change in developed land area during the historical period displayed variability but maintained an overall uniform distribution across all distance zones, with consistently lower growth rates inside the floodplain boundary. During the historical period, states along the Northeast Coast, including Rhode Island, Connecticut, Delaware, Maryland, and New Jersey, saw the highest development growth in zones situated farther away from the floodplain. In the Southeast Coast region, growth rates inside the floodplain were generally lower, while uniform growth was observed across other distance zones in states like Florida, Georgia, South Carolina, North Carolina, and Virginia. Across the South Central region, states such as Texas, Oklahoma, Kansas, and Arkansas displayed uniform percent change across all distance zones, with distinctively lower values inside the floodplain and in areas more than 2 km away from its boundary.

Simulations from 2019–2060 show new development occurring not only inside the floodplain but also across the other distance zones in all regions (Fig 6). Under this “business as usual” scenario, several states, including Washington, Nevada, Utah, Arizona, Wyoming, New Mexico, Texas, Georgia, Florida, South Carolina, Minnesota, New Hampshire, Massachusetts, New Jersey, Maryland, are projected to experience growth of over 20% in developed land area within the 100-year floodplain. In contrast, a growth rate of 20% or greater is anticipated only in Utah within the 0–250 m distance zone, in New Mexico across the 750–1000, 1000–1250, 1250–1500, 1500–1750, and 1750–2000 m zones, and in Texas across the 1500–1750, 1750–2000, and beyond 2-km zones.

### 3.3. County trends and projections

**Fig 7:**
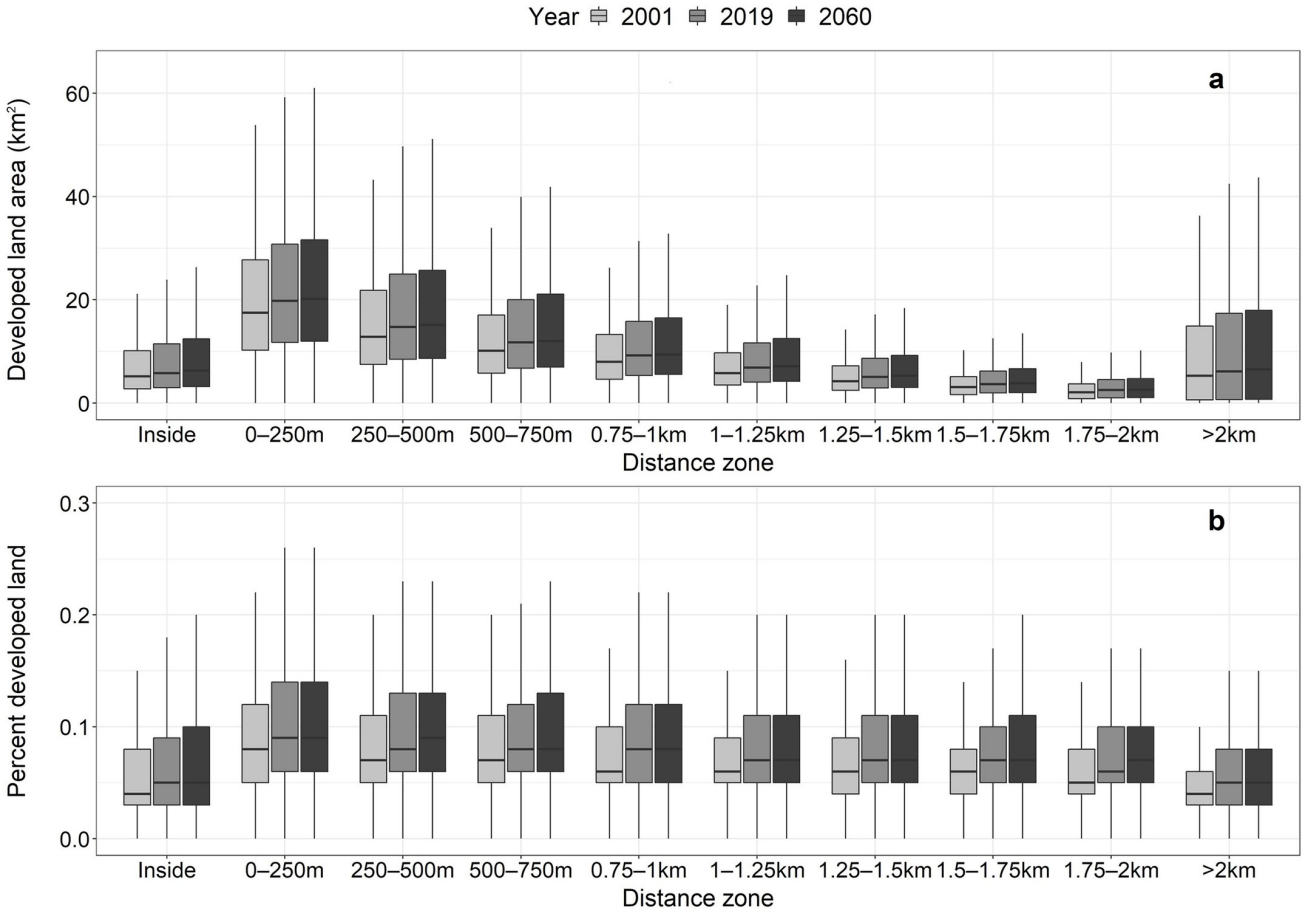
Distribution of total and percent developed land by distance zone and year (2001, 2019, 2060). (a) Total developed land area and (b) percent developed land (i.e., as a proportion of total land area in each zone) shown for each distance zone. Distributed values shown for the 2,330 U.S. counties included in this study. Total land area excludes open water bodies and permanently protected areas. Outliers removed for boxplot displays but retained in statistical analyses.

Our analysis of development patterns at the county-level revealed that mean amounts of developed land increased significantly between 2001 and 2019 both inside the floodplain and in all zones outside of the floodplain (Fig 7a; p < 0.05 two-sample unequal variance *t*-tests). However, between 2019 and 2060, our simulations projected a statistically significant increase in the mean amount of developed land within the floodplain only (Fig 7a; p < 0.05 two-sample unequal variance *t*-tests). At all time steps (2001, 2019, 2060), we found the average area (km^2^) of development in the 0–250 m zone to be significantly greater than the mean amounts inside the floodplain and in each distance zone beyond 250 m of the floodplain (Fig 7a and S2 Table; p < 0.001 two-sample unequal variance *t*-tests).

**Fig 8:**
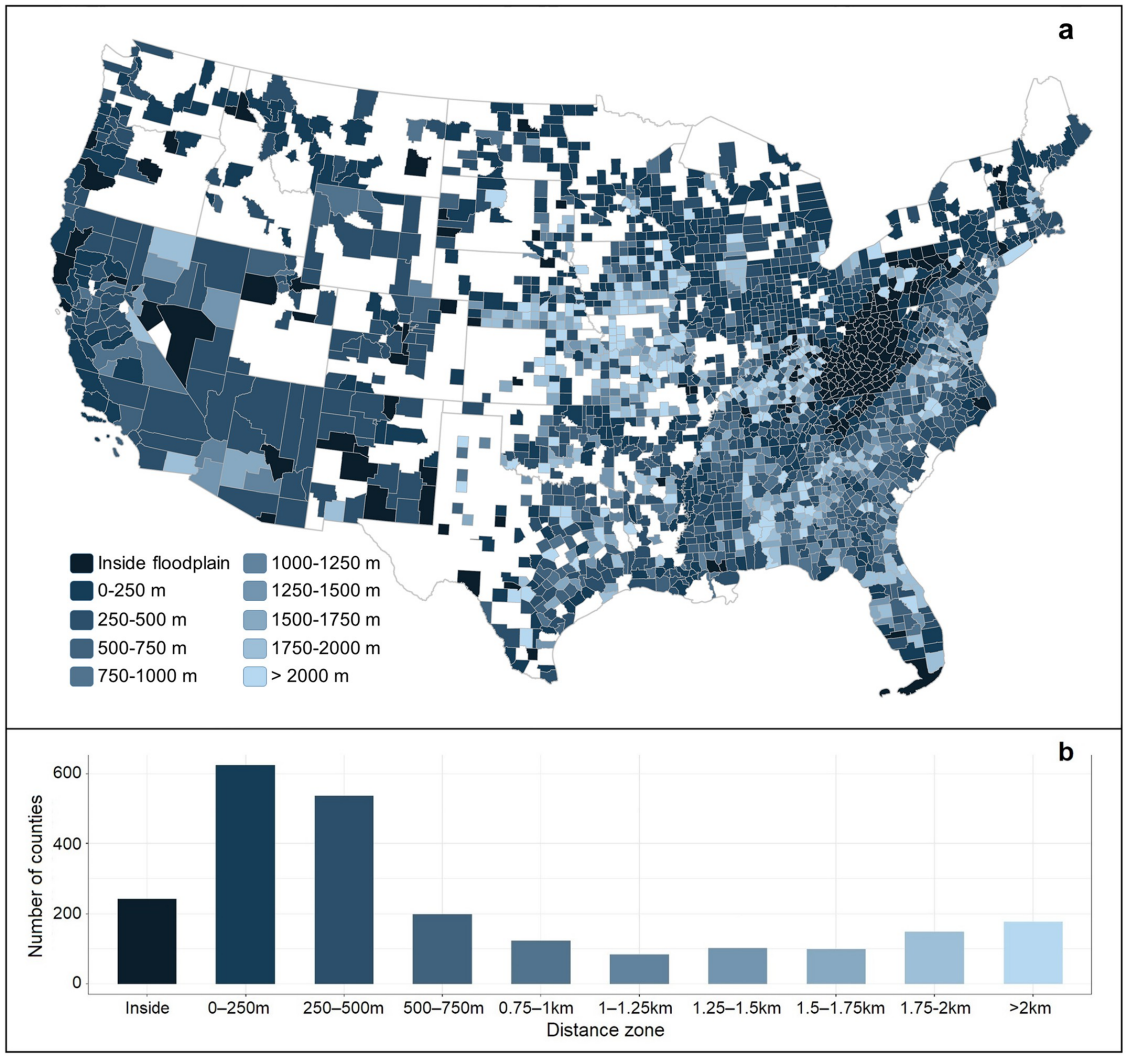
(a) Distance zone in each county with the highest percentage of developed land (i.e., as a proportion of total land area in each zone) in 2019, and (b) the number of counties with the highest percentage of developed land in each zone. Areas in white are not fully mapped by FEMA and excluded from the study. State and county boundaries are public domain data sourced from the U.S. Census Bureau’s TIGER/Line Shapefiles. All other data produced by the authors.

We found the average percent developed land to be highest in the 0–250 m zone at all time steps, and a general trend of increasing percent developed land with a zone’s proximity to the floodplain (Fig 7b). In 27% (629) of counties, the highest values of percent developed land in 2019 occurred within the 0–250 m zone (Figs 8a, 8b and S6). In half of all counties, the highest percentages of developed land are in the 0–250 m and 250–500 m zones (Figs 8a, 8b and S6b, S6bc). For 10% (242) of the counties, we found the highest percentages of developed land in the “inside floodplain” zone (Fig 8a, 8b and S6a). Many of these latter counties are concentrated in the Ridge and Valley region of the Appalachian Mountains extending from western North Carolina through Kentucky and West Virginia to Pennsylvania (Fig 8a and S6a Fig).

## 4. Local case studies: Beaufort County, NC, and the North Fork Kentucky River, KY

In this section, we present two case studies to illustrate that the broader CONUS-scale assessment is also relevant at local scales. Case study locations were selected for their contrasting terrains, flooding conditions, and availability of relevant data for this analysis. In Beaufort County, NC, and the North Fork Kentucky River (NFKR) catchment (Hydrologic Unit Code 12 051002010306), KY, we analyzed the distribution of buildings relative to the 100-year floodplain and to flood extents resulting from extreme weather events in 2018.

Beaufort County, a low-lying area characterized by low-density development in eastern North Carolina, was severely impacted by Hurricane Florence in 2018. The county experienced particularly intense damage, with residents reporting some of the highest levels of destruction across North Carolina [46, 47]. The hurricane and its associated coastal storm surge and rainfall caused extensive flooding across the Coastal Plain of North and South Carolina with 34 reported fatalities and an estimated $24 billion in damages to homes, structures and other property [48]. Also in 2018, excessive rainfall in the Central Appalachians resulted in elevated river flows and flash flooding along the North Fork Kentucky River [49]. The rugged terrain in this region limits development to relatively flat lands nearest the river that are susceptible to significant flooding, including residential areas and roads. The flooding events led to power outages, boil water advisories, water rescues, and structural integrity inspections [50].

### 4.1. Approach to local case studies

In both case study locations, we identified all building structures using the publicly available Microsoft building footprints dataset [51]. These building footprints, generated from satellite imagery using computer vision algorithms, are available in vector shapefile format across the United States. We then overlaid these building locations with datasets predicting flood extents during extreme events in 2018. For Beaufort County, NC, we used predicted flood extents from Hurricane Florence, generated by [52] using a machine learning algorithm (random forest) to delineate 10-meter radar imagery from before and after the storm. For the NFKY case, we used flood-inundation maps generated by [49, 53] through hydraulic modeling, also available in vector shapefile format. These models estimate inundation areas by simulating water flow, sediment transport, and floodplain dynamics based on flood-event water levels from USGS streamgages. We then used the building footprints and inundation extents to quantify the total number and proportion of buildings inside flood extents (referred to as flooded), outside flood extents (non-flooded), and within each of our ten analysis zones.

### 4.2. Observed trends in local case studies

In both the county and the catchment, we found that over 75% of buildings are situated inside the floodplain and within 0.5 km of the floodplain boundary. Specifically, Beaufort County had 31% of its buildings within the floodplain, 29% within the 0–250 m zone, and 15% in the 250–500 m zone (Section 3 in S1 File). In the NFKR catchment, 24% of all buildings are within the floodplain, 46% in the 0–250 m zone, and 15% in the 250–500 m zone (Section 3 in S1 File).

**Fig 9:**
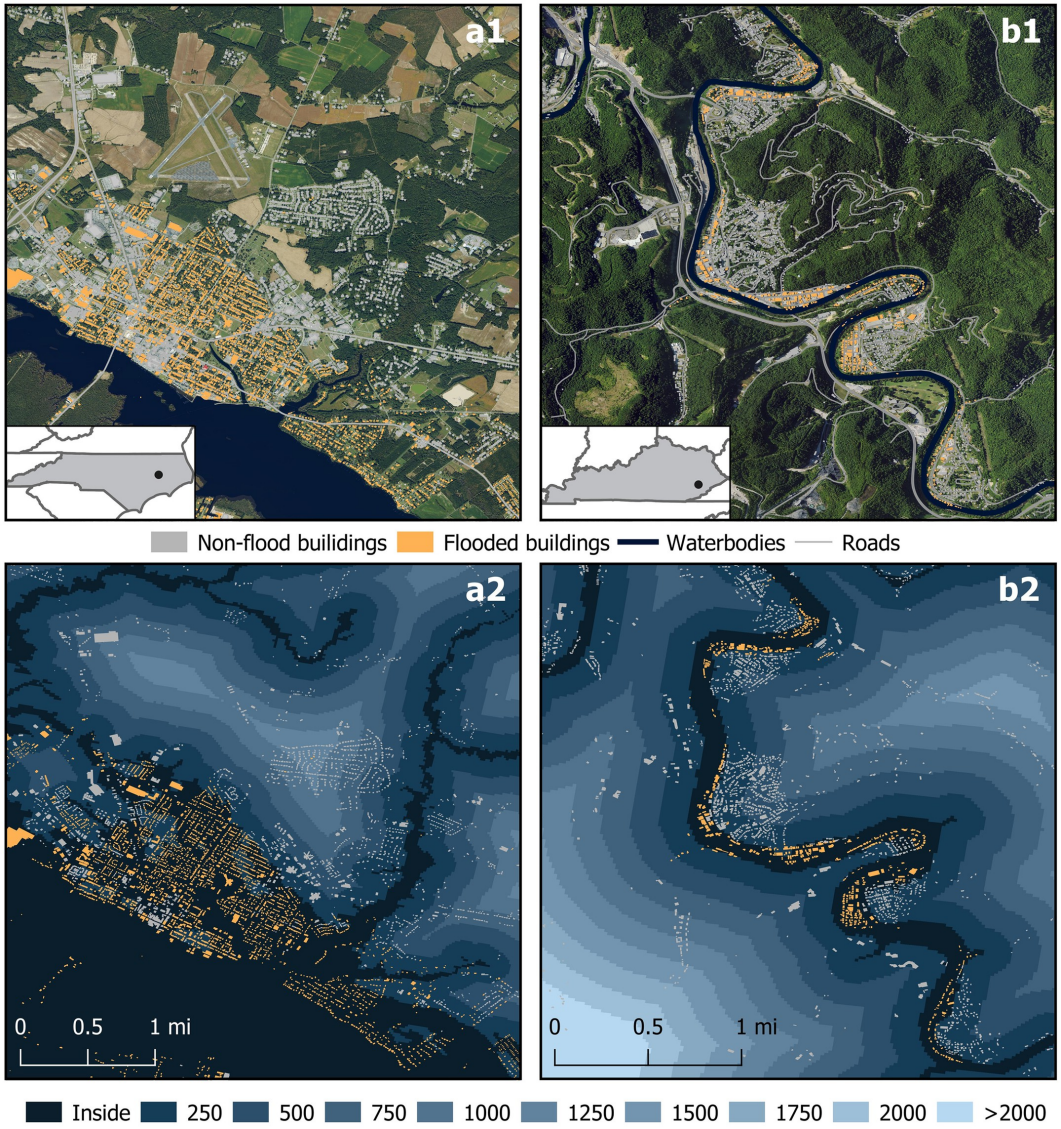
Detailed views of the case study areas showing buildings inside flood extents (referred to as flooded [yellow]) and outside flood extents (non-flooded [grey]) in the cities of (a) Washington, NC (Beaufort County), and (b) Hazard, KY (North Fork Kentucky River catchment). (a1) Aerial view of Washington, NC, and surrounding forest and farmland along the Pamlico River, and (a2) ten distance zones, superimposed with flooded and non-flooded buildings resulting from coastal flooding during 2018’s Hurricane Florence [52]. (b1) Aerial view of Hazard, KY, and surrounding mountains along the North Fork Kentucky River, and (b2) ten distance zones, overlaid with flooded and non-flooded buildings resulting from riverine flooding in 2018 [53]. Building structures as depicted by the publicly available Microsoft building footprints dataset [51]. Aerial images in a1 and b1 maps are public domain data sourced from the U.S. Department of Agriculture’s National Agriculture Imagery Program. State boundaries and roads are public domain data sourced from the U.S. Census Bureau’s TIGER/Line Shapefiles. Building footprints are available to use and share freely under the Open Data Commons Open Database License (ODbL). All other data produced by the authors.

We also found substantial proportions of flooded buildings located beyond the regulatory floodplain boundary as a result of these 2018 flood events. For example, during Hurricane Florence, whereas a majority of flooded buildings (63%, or 5,490 buildings) in Beaufort County were within the floodplain, another 23% of flooded structures (1,989) were located within the 0–250 m zone (Section 3 in S1 File), an area FEMA flood maps designate as having "minimal" flood risk. Washington, NC, the county seat of Beaufort County located at the confluence of the Pamlico and Tar Rivers near the Pamlico Sound (Fig 9a1), experienced unprecedented flood damages from rainfall and coastal storm surge that inundated buildings primarily in the floodplain and within 250 m of the floodplain boundary (Fig 9a2).

In the NFKR catchment, we found that a majority of flooded buildings (87%, or 490 buildings) exposed to the 2018 flood event were located within the floodplain, with the remaining 12% (70 buildings) located in the 0–250 m zone (Section 3 in S1 File). The City of Hazard in Perry County (Fig 9b1) was one of the most severely impacted locations [49] in the catchment, having most of its buildings located on the inside of multiple river bends, either within the floodplain or within 250 m of the floodplain boundary (Fig 9b2). Overall, these case studies demonstrate the risk management challenges posed by the SDP across different geographies.

## 5. Discussion

Our findings consistently reveal a disproportionate concentration of development within the first 250 m immediately adjacent to the 100-year floodplain, a trend that our projections indicate will persist through 2060 if no measures are taken to reduce flood exposure. We observed evidence for the safe development paradox at the national level (24% of all developed land is in the 0–250 m distance zone), state level (9–24% of all developed land is in the 0–250-m zone), and county level (an average of 9%) through time (2001–2019, 2019–2060). Our two local-scale case studies in Beaufort County, NC, and the North Fork Kentucky River Catchment, KY, further illustrate how a substantial number of buildings impacted by flood events are located adjacent to the regulatory 100-year floodplain boundary. Our results align with previous research, confirming that a significant portion of human settlements and assets are located within designated high-risk zones [1, 27, 28]. However, our study highlights an additional concern: the clustering of development near the edge of the floodplain, despite flood risk persisting beyond this boundary. Our findings underscore the importance of considering the broader impacts of flood management policies on development patterns, as they can extend beyond the areas they are designed to protect [25].

During the 2001–2019 period, we observed spatial variations in development growth rates. Specifically, regions with a high proportion of land in the 100-year floodplain, such as the Southeast Coast, experienced relatively slower development growth rates within the floodplain compared to areas outside of it (Fig 6). This pattern aligns with previous studies that have shown a greater responsiveness to flood management policies in regions with higher exposure to flooding, resulting in a tendency to avoid development in regulatory hazard zones [21, 54, 55]. By 2060, future projections indicate an 18% increase (4,840 km^2^) in developed land area within the 100-year floodplain relative to 2019 (Fig 3a and Section 2 in S1 File). This finding aligns with earlier studies projecting a 13.3% to 15.6–15.8% rise in the U.S. population residing within the 100-year floodplain by 2050 [1]. However, previous studies overlooked areas beyond regulatory hazard zones (i.e., outside of the 100-year floodplain). We found that currently 23.7% of all developed land in the CONUS is located within 250 m of the floodplain, and projections indicate an increase to 24.1% by 2060. Moreover, the total developed land area in the 0–250 m zone (89,080 km^2^) is over three times the developed area inside the floodplain (26,938 km^2^), approximately 1.4 times the developed land area in the adjacent 250–500 m distance zone (64,855 km^2^), and twice the amount of developed land in the zone outside 2 km from the floodplain boundary (44,816 km^2^; Fig 3a and Section 2 in S1 File).

Similar to national trends, low-lying coastal states with relatively flat topographies and a large proportion of land dominated by floodplains, such as Louisiana (42% of total land area in the 100-year floodplain), Florida (36%), and Texas (15%), exhibit well-distributed growth across all distance zones beyond the floodplain (Fig 5 and S1 Table). This could be attributed to the greater availability of suitable land for urban development, with fewer constraints like steep topography, resulting in sprawling development across all distance zones beyond the floodplain boundary. In contrast, states characterized by mountainous topographies tend to rely on limited flat areas, typically near river networks, for settlement. This pattern is most evident in states like Montana (3%), Idaho (4%), Washington (4%), and Oregon (4%), which have the lowest proportion of total land area in the 100-year floodplain, yet show some of the highest proportions of developed land in the 0–250 m distance zone (Fig 5 and S1 Table).

The development patterns we observed demonstrate the complex interplay between a preference for proximity to water bodies and avoidance of high-risk areas. On one hand, population growth and development are strongly associated with ocean and lake coastlines, which contribute to economic productivity and quality of life [56, 57]. On the other hand, the regulatory 100-year floodplain discourages urban development in high-risk areas [21, 54], as people seek to mitigate potential damage and avoid the financial burden associated with development requirements such as dry-proofing and elevating infrastructure [29], as well as mandatory flood insurance [15, 18]. As of 2024, however, FEMA’s NFIP regulations do not require disclosure of flood risk to property buyers in areas beyond the 100-year floodplain boundary, such as the 500-year floodplain, which has a 0.2% chance of flooding in any given year or a 6% chance of flooding over a 30-year mortgage [58]. We posit that the binary categorization of risk zones (i.e., inside or outside of the 100-year floodplain) often leads to the perception that properties just outside of the floodplain bear minimal to no risk, regardless of the actual risk [13, 29]. As a result, development concentrates in areas adjacent to the floodplain, where the perceived lower risk may be misleading.

### 5.1. Policy implications

Residents acquiring properties within the floodplain (with a federally backed or regulated lender) must comply with development requirements and purchase flood insurance, thereby better protecting them and their property from flood damage [17]. Paradoxically, residents who choose to stay outside of regulatory hazard zones but still opt for locations near water bodies may perceive their property as “safe” and therefore are less likely to purchase flood insurance or follow strict building requirements [59, 60]. In this study, we present empirical evidence of concentrated development just beyond the regulatory floodplain, emphasizing the importance of comprehensive studies that recognize regulatory floodplains as a case of the SDP. Our findings indicate that regulatory flood hazard zones, with their binary representation of risk as "inside" or "outside," contribute to structurally embedded risky behavior [17, 61]. In response to this problem, previous assessments of FEMA’s mapping efforts have proposed a shift towards property-specific flood risk assessments or spatially continuous estimates of flood hazard [5, 25, 61–64], aiming to provide a more comprehensive understanding of flood risk beyond the 100-year floodplain. A recent advance in this direction is FEMA’s new pricing methodology, Risk Rating 2.0. This methodology incorporates spatially varying flood risk factors, such as flood frequency and distance to water, to more accurately depict property flood risk. However, Risk Rating 2.0 is limited to the designated 100-year floodplain and exclusively influences property flood insurance rates. Additionally, various other flood risk mitigation strategies are viable, such as enhancing risk communication methods [29, 65] and utilizing other publicly accessible sources of reliable flood risk information that residents can consult (e.g., [66]). By effectively conveying the risks of flooding and promoting strategies to mitigate its adverse effects, overall damage can be significantly reduced. Additionally, increasing awareness about the risks associated with living in and around regulatory hazard zones, residents can better understand their potential flood likelihood and take appropriate measures to mitigate those risks.

Given converging factors such as concentrated development at the edge of the floodplain, inaccuracies in flood maps [23, 25], population growth in and around flood-prone areas [1, 34], and the escalating flood hazards associated with climate change [67], future exposure to flooding will likely surpass previous estimates for both people and infrastructure. These factors, when combined, increase the potential for significant damage and losses due to flooding events. While FEMA FIRMs delineate the 100-year floodplain, offering a static representation—a snapshot [13]—they do not account for the changes in flood hazard that are projected in the years or decades to come. This limits their utility for informing future development or adaptive management decisions. Recent modeling efforts, differing from FEMA’s current approach, account for climate change’s impact on combined fluvial, pluvial, and coastal flood hazards across space and time. These efforts have already revealed that properties currently situated just outside the 100-year floodplain are unlikely to remain unaffected in future decades [1, 67, 68]. Moreover, coastal communities face disproportionate risks due to increased frequency in storm events, tidal flooding, and sea-level rise [69–72].

### 5.2. Future research and study limitations

Our study provides novel insights into how floodplain regulation may paradoxically increase flood exposure and risk at the edge of the floodplain. However, there are additional considerations that could be addressed in future research. This study is limited to flood regulations in the U.S., though many other countries also use risk-based approaches to floodplain management. Definitions of “high-risk” flood zones vary across countries. For example, the U.S., Canada, Germany define high-risk zones as areas with a 1% annual probability of flooding [73, 74], while the United Kingdom uses a 1% threshold for riverine flooding and a 0.5% for coastal flooding [75]. Future research could explore the presence of the safe development paradox in floodplain regulation across the globe. Attention could also be given to how authority differs by country. In the U.S., floodplain management is relatively decentralized, with federal agencies like FEMA providing guidelines, but significant authority resides with local and state governments. In contrast, the Netherlands, given its smaller size and low-lying geography, adopts a more centralized approach with coordinated national strategies and a stricter 0.01% annual probability standard [76].

Our study examines amount of developed land within and surrounding floodplains but does not delineate between types of development (e.g., residential, commercial, industrial). Research on flood risk management highlights the clear disparities faced by socially vulnerable populations—particularly female, economically disadvantaged, Black, and Hispanic individuals—who often bear a disproportionate burden of flood risk [77–80]. While past studies have primarily focused on documenting the communities that experience higher exposure within the regulatory floodplain [27, 79, 80] or those directly affected by flood events [77, 78], there remains an opportunity for future work to comprehensively assess the socio-economic background of communities residing immediately adjacent to the floodplain boundary. Understanding the socio-economic context of at-risk communities can provide valuable insights into the unintended consequences of flood management policies and interventions that may disproportionately harm vulnerable populations [5]. Future research could also consider how localized economic drivers and zoning regulations shape settlement patterns in relation to designated high-risk zones, providing a more in-depth understanding of development in areas with disproportionate exposure. Further assessments may also consider the impacts of climate change and sea-level rise on the spatial footprint of flood risk and how different communities may respond and adapt to increasing flood risks in the future [34, 80]. By incorporating socio-economic factors and considering the evolving impacts of climate change, future research can contribute to more equitable and effective flood risk management approaches.

## 6. Conclusions

In this study, we examined historical and future development patterns in relation to the regulatory 100-year floodplain. Our findings provide valuable insights into the "safe development paradox," where efforts to reduce flood risk can reshape land use decisions and paradoxically lead to increased exposure through the concentration of development in areas immediately adjacent to the regulatory floodplain boundary. We observed a consistent pattern of development concentration within the 0–250 m distance zone from the floodplain at the national, state, and county levels. This trend of increasing development near floodplain boundaries reflects a preference for proximity to water bodies and simultaneous avoidance of the 100-year floodplain, revealing the U.S. regulatory floodplain as a case of the safe development paradox. The binary categorization of risk zones, with properties either inside or outside the 100-year floodplain, often leads to the perception that properties just outside of the floodplain are safe from flooding risks. This perception can be misleading and discourages residents from purchasing flood insurance or following strict building requirements, potentially putting them at greater risk during flood events. Our study underscores the limitations of current floodplain maps in effectively communicating flood risk, particularly with the dynamic nature of flood hazards. A forward-looking approach to floodplain management should prioritize spatially continuous estimates of flood hazard rather than a simplified binary representation of risk. Our approach to examining the SDP phenomenon at a large scale can be applied across various regulatory contexts to understand how designated flood zones influence development patterns worldwide.

## Supporting information

S1 Fig Overall model accuracy is presented as (A) the share of simulation successes and errors and (B) the proportion of error associated with quantity and allocation disagreement for the validation reference period (i.e., 2001–2008). FUTURES framework simulates patterns of new development and assumes no further growth in already developed areas. To capture this model assumption, the proportion of initially developed areas (i.e., 2008) is partitioned (7.99% of the landscape; A). Null successes refer to locations where the model correctly simulates no change between the simulation period (2009–2019). Hits refer to locations where the model correctly simulates observed change. False alarms refer to locations where the model simulates change where no change was observed. Misses refer to locations where the model did not simulate change where it was observed. Allocation error measures the share of the landscape where the location of simulated change does not match observed change. Quantity error measures the disagreement between simulated and observed amounts of change. Values estimated from 20 stochastic urban growth simulations computed across the CONUS (permanently protected areas and water bodies were excluded).
(TIF)

S2 Fig Spatial distribution of simulation accuracy estimated by figure of merit (FoM). FoM quantifies the statistical correspondence between observed and simulated land change. FoM is calculated at 10 x 10 km grid cells and derived from 20 stochastic urban growth simulations computed across the CONUS (permanently protected areas and water bodies were excluded). State boundaries are public domain data sourced from the U.S. Census Bureau’s TIGER/Line Shapefiles. All other data produced by the authors.
(TIF)

S3 Fig Spatial distribution of simulation accuracy estimated by mean allocation error. Allocation error evaluates whether the locations of simulated and observed land changes differ. Mean allocation error is calculated at 10 x 10 km grid cells and derived from 20 stochastic urban growth simulations computed across the CONUS (permanently protected areas and water bodies were excluded). State boundaries are public domain data sourced from the U.S. Census Bureau’s TIGER/Line Shapefiles. All other data produced by the authors.
(TIF)

S4 Fig Spatial distribution of simulation accuracy estimated by mean quantity error. Quantity error evaluates whether the total amount of simulated and observed land changes differ, highlighting locations where the model under or over predicted change. Mean quantity error is calculated at 10 x 10 km grid cells and derived from 20 stochastic urban growth simulations computed across the CONUS (permanently protected areas and water bodies were excluded). State boundaries are public domain data sourced from the U.S. Census Bureau’s TIGER/Line Shapefiles. All other data produced by the authors.
(TIF)

S5 Fig Simulation accuracy estimated by figure of merit and plotted by development density. Values averaged from 20 stochastic urban growth simulations computed across the CONUS (permanently protected areas and water bodies were excluded).
(TIF)

S6 Fig Percentage of developed land (i.e., as a proportion of total land area in each zone) in 2019 occuring in each of the ten distance zones (a–j), mapped by county across the CONUS. Areas in the maps with no shading are counties not fully mapped by FEMA and excluded from the study. State and county boundaries are public domain data sourced from the U.S. Census Bureau’s TIGER/Line Shapefiles. All other data produced by the authors.
(TIF)

S1 Table State-level estimates (2001, 2019) and projections (2060) of developed land area by distance zone from the 100-year floodplain. Values from the 2,330 U.S. counties included in this study with available FEMA floodplain maps. Table attached to manuscript.
(XLSX)

S2 Table County-level ranking of concentration of developed land immediately adjacent (0–250 m distance zone) to the 100-year floodplain. Values calculated for 2019. Table attached to manuscript.
(XLSX)

S1 File Appendix. (DOCX)
