# Supplementary figures and images for: The safe development paradox of the United States regulatory floodplain
Source: PLoS One. 2024 Dec 31;19(12):e0311718. doi: 10.1371/journal.pone.0311718 (PMC11687735; doi:10.1371/journal.pone.0311718)

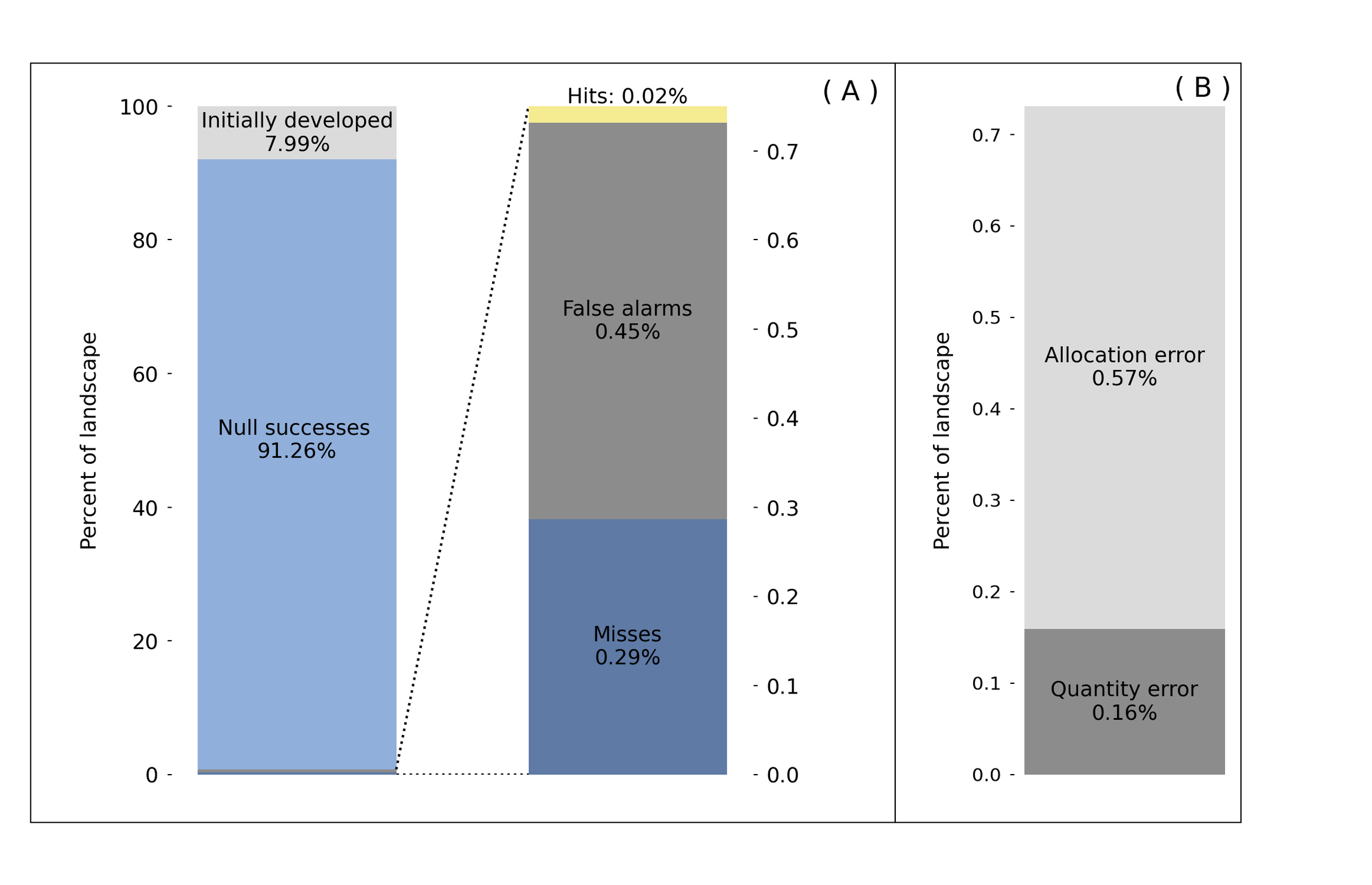

Supplement: S1 Fig — Overall model accuracy is presented as (A) the share of simulation successes and errors and (B) the proportion of error associated with quantity and allocation disagreement for the validation reference period (i.e., 2001–2008). FUTURES framework simulates patterns of new development and assumes no further growth in already developed areas. To capture this model assumption, the proportion of initially developed areas (i.e., 2008) is partitioned (7.99% of the landscape; A). Null successes refer to locations where the model correctly simulates no change between the simulation period (2009–2019). Hits refer to locations where the model correctly simulates observed change. False alarms refer to locations where the model simulates change where no change was observed. Misses refer to locations where the model did not simulate change where it was observed. Allocation error measures the share of the landscape where the location of simulated change does not match observed change. Quantity error measures the disagreement between simulated and observed amounts of change. Values estimated from 20 stochastic urban growth simulations computed across the CONUS (permanently protected areas and water bodies were excluded). (TIF) [file pone.0311718.s001.tif]

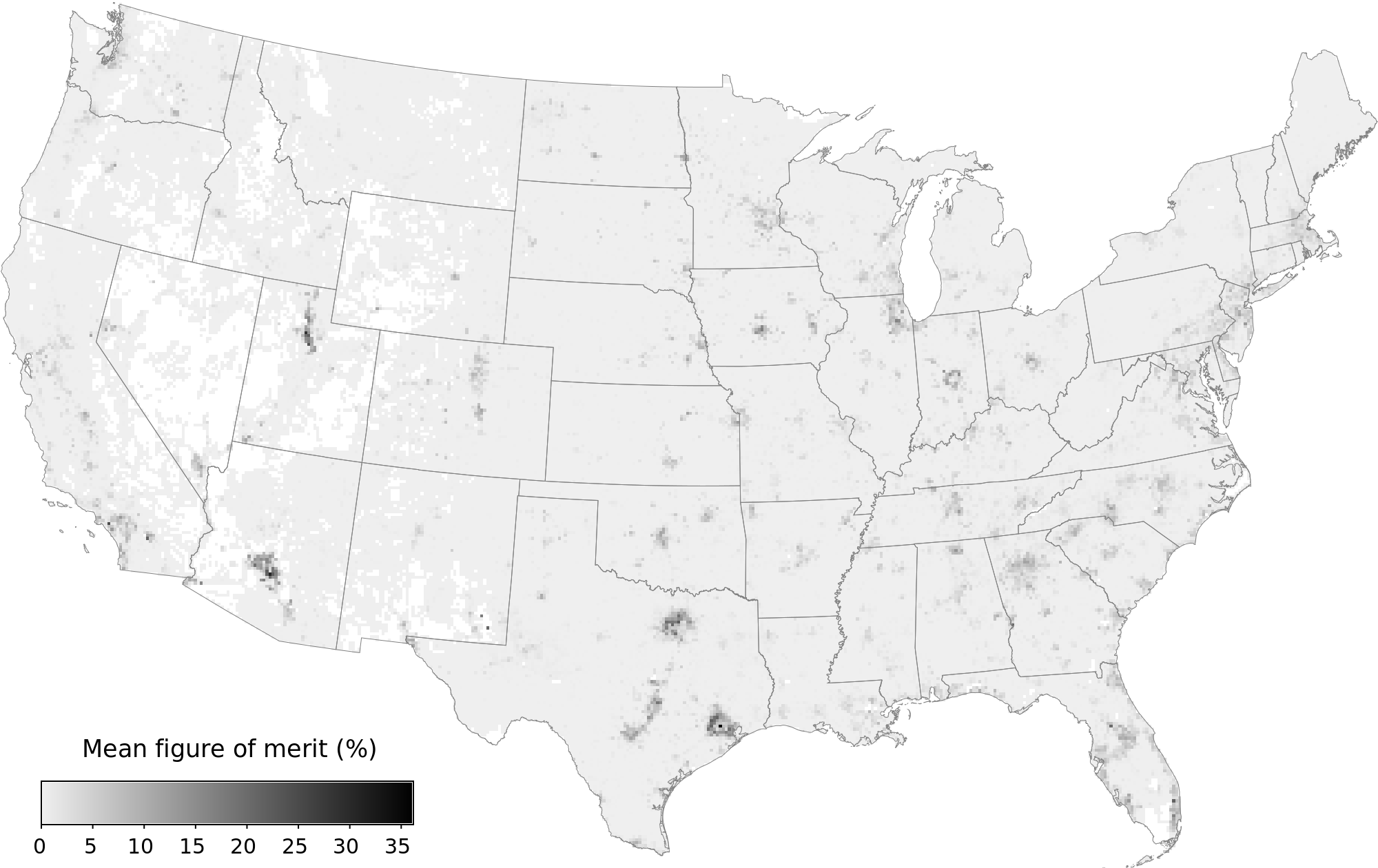

Supplement: S2 Fig — FoM quantifies the statistical correspondence between observed and simulated land change. FoM is calculated at 10 x 10 km grid cells and derived from 20 stochastic urban growth simulations computed across the CONUS (permanently protected areas and water bodies were excluded). State boundaries are public domain data sourced from the U.S. Census Bureau’s TIGER/Line Shapefiles. All other data produced by the authors. (TIF) [file pone.0311718.s002.tif]

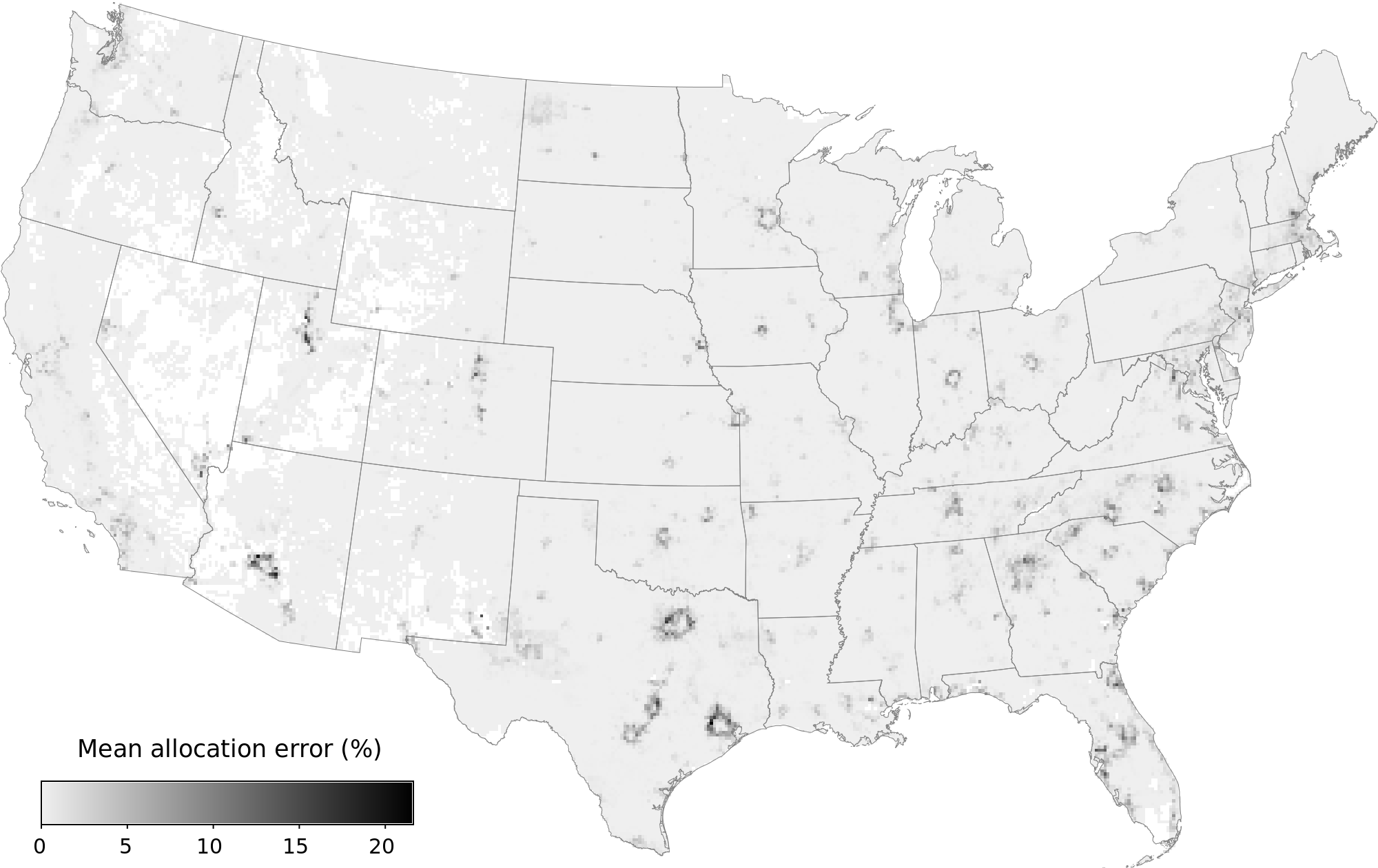

Supplement: S3 Fig — Allocation error evaluates whether the locations of simulated and observed land changes differ. Mean allocation error is calculated at 10 x 10 km grid cells and derived from 20 stochastic urban growth simulations computed across the CONUS (permanently protected areas and water bodies were excluded). State boundaries are public domain data sourced from the U.S. Census Bureau’s TIGER/Line Shapefiles. All other data produced by the authors. (TIF) [file pone.0311718.s003.tif]

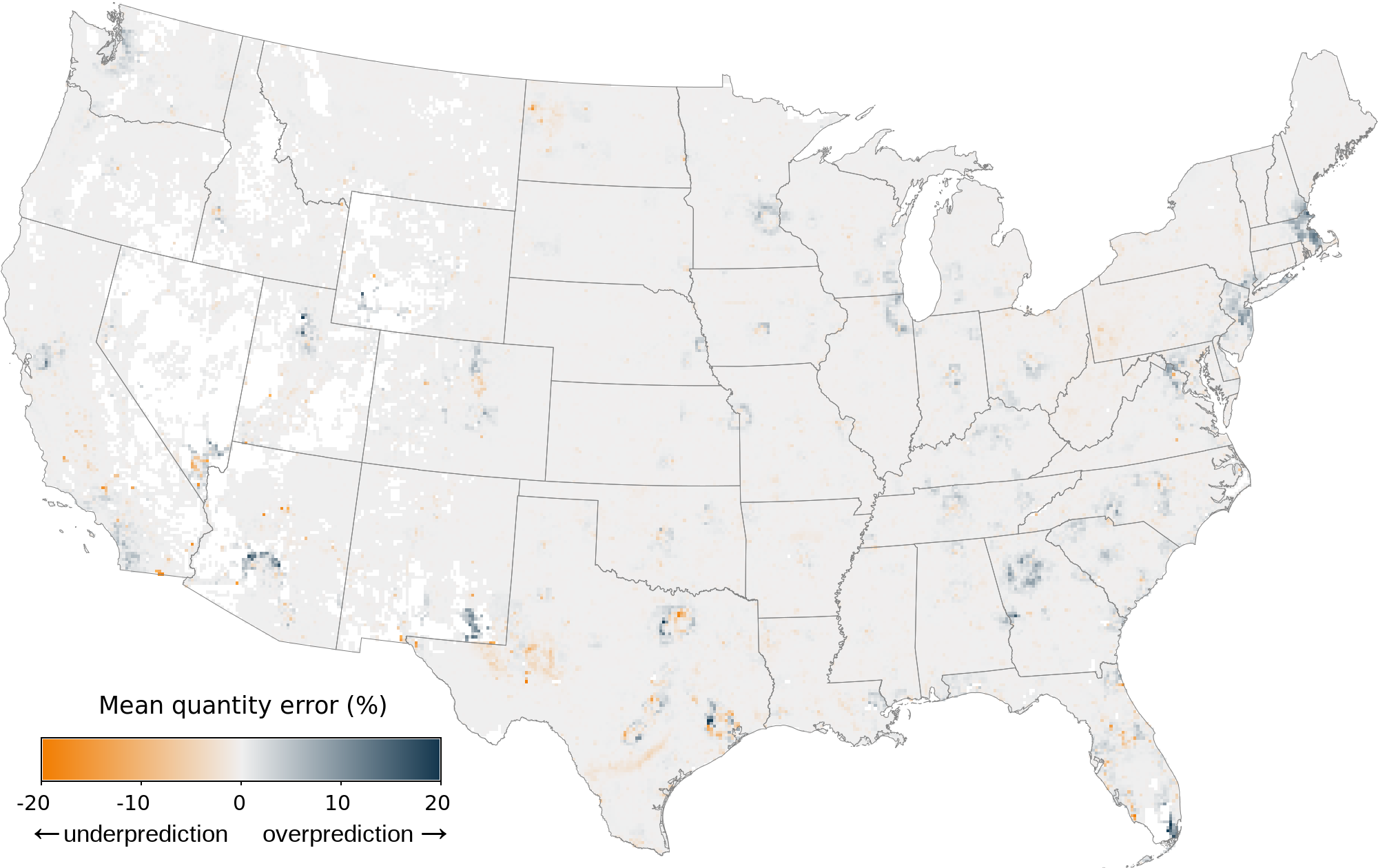

Supplement: S4 Fig — Quantity error evaluates whether the total amount of simulated and observed land changes differ, highlighting locations where the model under or over predicted change. Mean quantity error is calculated at 10 x 10 km grid cells and derived from 20 stochastic urban growth simulations computed across the CONUS (permanently protected areas and water bodies were excluded). State boundaries are public domain data sourced from the U.S. Census Bureau’s TIGER/Line Shapefiles. All other data produced by the authors. (TIF) [file pone.0311718.s004.tif]

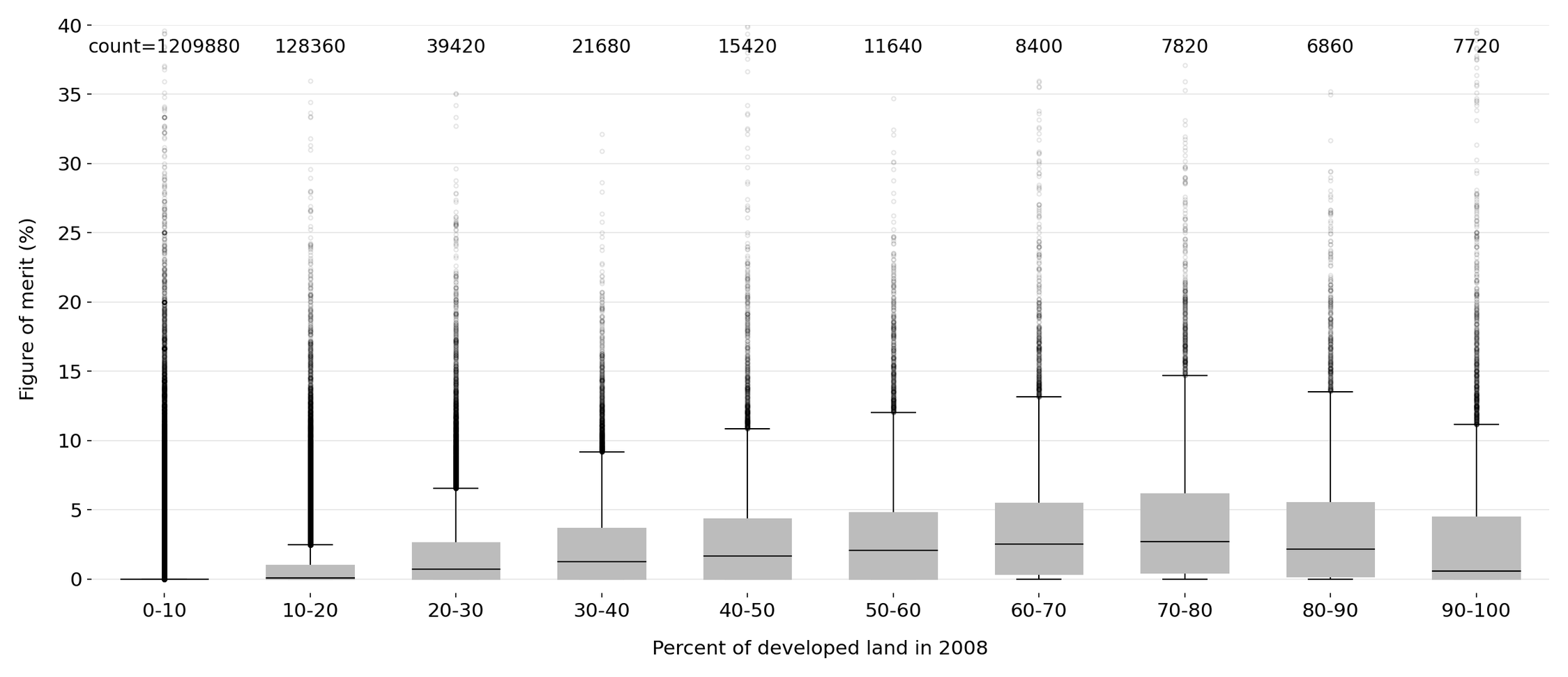

Supplement: S5 Fig — Values averaged from 20 stochastic urban growth simulations computed across the CONUS (permanently protected areas and water bodies were excluded). (TIF) [file pone.0311718.s005.tif]

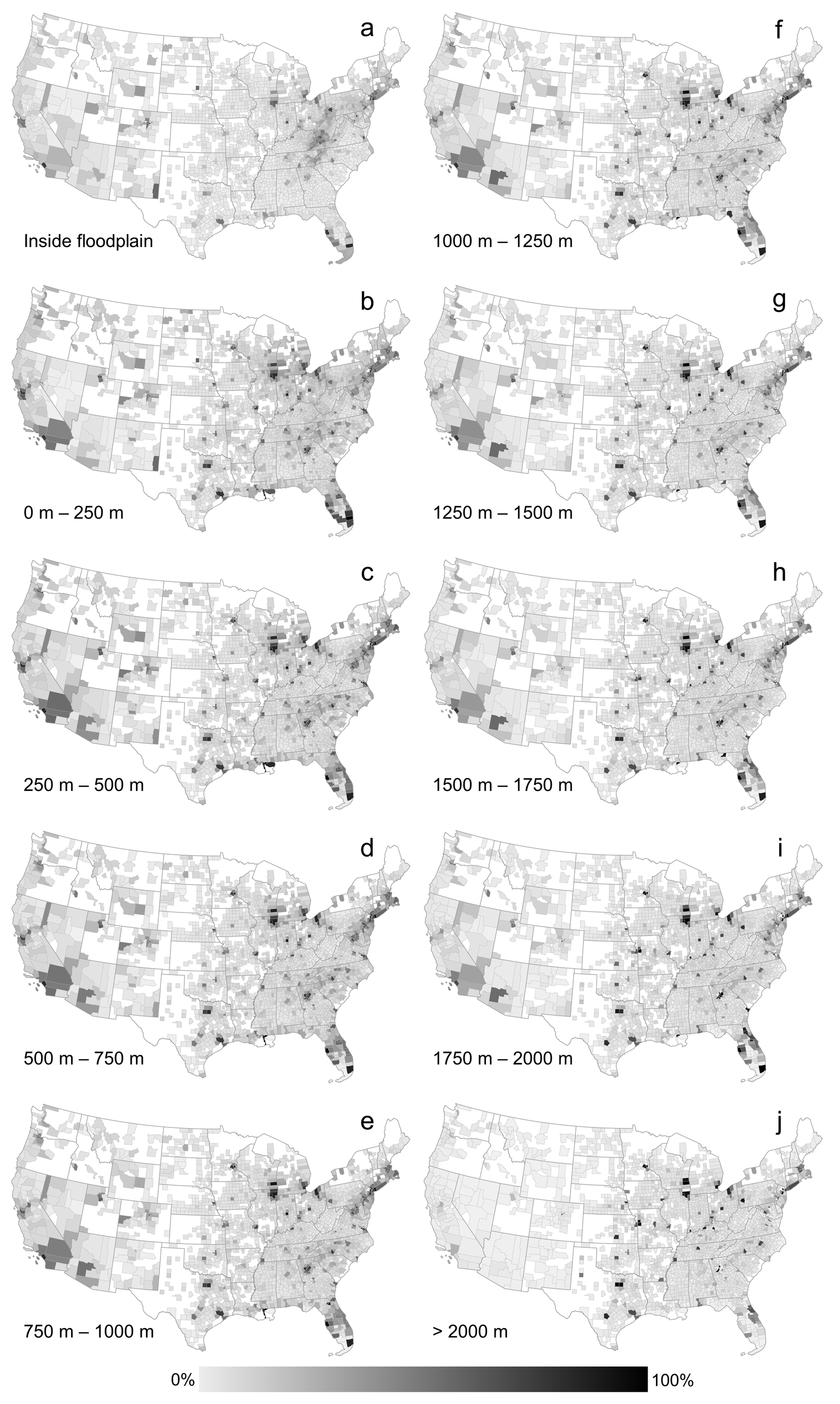

Supplement: S6 Fig — Percentage of developed land (i.e., as a proportion of total land area in each zone) in 2019 occuring in each of the ten distance zones (a–j), mapped by county across the CONUS. Areas in the maps with no shading are counties not fully mapped by FEMA and excluded from the study. State and county boundaries are public domain data sourced from the U.S. Census Bureau’s TIGER/Line Shapefiles. All other data produced by the authors. (TIF) [file pone.0311718.s006.tif]
