# Supplementary material for: The safe development paradox of the United States regulatory floodplain
Source: PLoS One. 2024 Dec 31;19(12):e0311718. doi: 10.1371/journal.pone.0311718 (PMC11687735; doi:10.1371/journal.pone.0311718)
Supplement: S1 File — (DOCX) [file pone.0311718.s009.docx]

**The safe development paradox of the United States regulatory floodplain**

Georgina M. Sancheza,*, Margaret A. Lawrimore^a^, Anna Petrasova^a^, John B. Vogler^a^, Elyssa L. Collins^a^, Vaclav Petras^a^, Truffaut Harper^a^, Emma J. Butzler^a^, Ross K. Meentemeyer^a,b^.

^a^Center for Geospatial Analytics, North Carolina State University, 2800 Faucette Dr., Raleigh, NC 27695, USA

^b^Department of Forestry and Environmental Resources, North Carolina State University, 2820 Faucette Dr., Raleigh, NC 27695, USA

*Corresponding author: Georgina M. Sanchez.

Email: [gmsanche@ncsu.edu](mailto:gmsanche@ncsu.edu)

# Supporting information

## 1. Land change model parameterization and validation

**Table 1.** Future Urban-Regional Environment Simulation (FUTURES) submodel predictor variables, description, and source.

| Submodel | Predictor | Description | Base data | Year(s) | Data source |
| --- | --- | --- | --- | --- | --- |
| **POTENTIAL** | | | | | |
|  | Land cover | Land classified as developed, forest, agricultural, water, wetland | NLCD | 2001, 2011, 2016, 2019 | Homer et al., 2020; Dewitz, 2021 |
|  | Roads | Euclidean distance to roads | TIGER/Line Shapefiles | 2019 |  |
|  | Topography | Slope | NED | 2011 | USGS, 2017 |
|  | Crop production | Average crop production | Crop Productivity Index derived from SSURGO | 2021 | USDA, 2021 |
|  | Multilevel structure | County and metropolitan statistical area boundaries | TIGER/Line Shapefiles | 2019 | US Census Bureau, 2019 |
|  | Development pressure | Number of nearby developed pixels within search distance and weighted by distance | Historical and projected development patterns | 2001-2050 | Homer et al., 2020; Dewitz, 2021; FUTURES simulations |
|  | Social Vulnerability Index | Census data to determine the social vulnerability of every census tract | SVI | 2018 | CDC 2018 |
|  | Proximity to the floodplain | Euclidean distance to the closest floodplain | 100-year floodplain | 2020 | FEMA, 2020 |
| **DEMAND** | | | | | |
|  | Population | Historical and projected population | SSP2 projections | 2001-2050 | Hauer, 2019; NVSS, 2021 |
|  | Development | Land classified as developed and undeveloped | NLCD | 2001, 2004, 2006, 2008, 2011, 2013, 2016, 2019 | Homer et al., 2020; Dewitz, 2021 |
| NLCD = National Land Cover Database (www.mrlc.gov); NED = National Elevation Database; TIGER = Topologically Integrated Geographic Encoding and Referencing (tigerweb.geo.census.gov); SSURGO = Soil Survey Geographic Database (www.nrcs.usda.gov); SVI = Social Vulnerability Index (www.atsdr.cdc.gov); FUTURES = FUTure Urban-Regional Environment Simulation; SSP2 = Shared Socioeconomic Pathways (Middle of the Road) | | | | | |

**Table 2.** Description of generalized linear mixed-effects POTENTIAL submodel (site suitability). Random effects vary by county and are calculated for intercept and development pressure.

| Fixed effects | Coefficient | Std. error |
| --- | --- | --- |
| Intercept | -2.960 | 0.012 |
| Social Vulnerability Index*** | -1.254 | 0.008 |
| Distance to roads (log transformed)*** | -0.106 | 0.001 |
| Distance to forest land (log transformed)*** | 0.264 | 0.002 |
| Distance to water (log transformed)*** | -0.221 | 0.002 |
| Percent herbaceous wetlands (1 km sq.)*** | -0.514 | 0.012 |
| Slope (log transformed)*** | 0.155 | 0.004 |
| Average crop production*** | 0.326 | 0.003 |
| Proximity to floodplain edge*** | 0.185 | 0.006 |
| Random effects | Variance | Std. dev. |
| County (intercept) | 0.939 | 0.969 |
| Development pressure | 0.773 | 0.879 |

*** p < 0.001

## 2. Supplementary statistics for national-level trends

**Table 3.** Total and percent developed land area by distance zone and year (2001, 2019, 2060) and developed land change over the 2001–2019 and 2019–2060 time periods for the CONUS. Values calculated from the 2,330 U.S. counties included in this study with available FEMA floodplain maps. Percent developed land values are normalized by total land area (i.e., as a proportion of the total zone area) in each zone.

| Distance zones | Total land area in km^2^ (*%) | Total 2001 developed land area in km^2^ (^†^%) | Total 2019 developed land area in km^2^ (^†^%) | Total 2060 developed land area in km^2^ (^†^%); SD in km^2^ | Developed land change 2001-2019 in km^2^ (^†^%) | Average developed land change 2019-2060 in km^2^ (^†^%) |
| --- | --- | --- | --- | --- | --- | --- |
| Inside | 418,108 (11) | 23,814 (6) | 26,938 (6) | 31,778 (8); 1,863 | 3,123 (13) | 4,840 (18) |
| 0–250 m | 608,944 (16) | 75,619 (12) | 89,080 (15) | 95,980 (16); 2,842 | 13,460 (18) | 6,900 (8) |
| 250–500 m | 473,366 (12) | 54,971 (12) | 64,855 (14) | 69,938 (15); 1,724 | 9,884 (18) | 5,083 (8) |
| 500–750 m | 377,185 (10) | 40,739 (11) | 48,178 (13) | 51,914 (14); 1,335 | 7,439 (18) | 3,736 (8) |
| 750–1000 m | 303,466 (8) | 30,079 (10) | 35,688 (12) | 38,413 (13); 1,102 | 5,609 (19) | 2,725 (8) |
| 1000–1250 m | 234,938 (6) | 21,302 (9) | 25,369 (11) | 27,265 (12); 892 | 4,067 (19) | 1,895 (7) |
| 1250–1500 m | 183,133 (5) | 15,309 (8) | 18,272 (10) | 19,587 (11); 732 | 2,963 (19) | 1,316 (7) |
| 1500–1750 m | 139,656 (4) | 10,808 (8) | 12,914 (9) | 13,821 (10); 601 | 2,106 (19) | 907 (7) |
| 1750–2000 m | 106,859 (3) | 7,572 (7) | 9,051 (8) | 9,670 (9); 478 | 1,478 (20) | 619 (7) |
| >2000 m | 1,011,461 (26) | 38,384 (4) | 44,816 (4) | 48,246 (5); 2,879 | 6,431 (17) | 3,431 (8) |

*% = percent of study system area; ^†^% = percent of distance zone area. SD = Standard Deviations; derived from 50 stochastic urban growth simulations. Total land area excludes open water bodies and permanently protected areas.

## 3. Supplementary statistics for case studies

**Table 4**. Total number and proportion of all buildings and all flooded buildings by distance zone attributed to a) coastal flooding associated with Hurricane Florence across Beaufort County, NC, and b) riverine flooding on February 10–11, 2018, in the North Fork Kentucky River (KY) catchment.

| Distance zones | Number of buildings  (% of all buildings) | Number of flooded buildings (% of all flooded buildings) | % of buildings in zone that are flooded |
| --- | --- | --- | --- |
| **Beaufort County** | |  |  |
| Inside floodplain | 9396 (30.8) | 5490 (62.9) | 58.4 |
| 0–250 m | 8889 (29.2) | 1989 (22.8) | 22.4 |
| 250–500 m | 4448 (14.6) | 449 (5.1) | 10.1 |
| 500–750 m | 2648 (8.7) | 246 (2.8) | 9.3 |
| 750–1000 m | 1874 (6.1) | 182 (2.1) | 9.7 |
| 1000–1250 m | 1078 (3.5) | 105 (1.2) | 9.7 |
| 1250–1500 m | 525 (1.7) | 68 (0.8) | 13.0 |
| 1500–1750 m | 387 (1.3) | 49 (0.5) | 12.7 |
| 1750–2000 m | 226 (0.7) | 24 (0.3) | 10.6 |
| >2000 m | 1016 (3.3) | 130 (1.5) | 12.8 |
| **North Fork KY River** | |  |  |
| Inside floodplain | 966 (23.7) | 490 (87) | 49.2 |
| 0–250 m | 1886 (46.2) | 70 (12.4) | 3.7 |
| 250–500 m | 593 (14.5) | 1 (0.18) | 0.02 |
| 500–750 m | 211 (5.2) | 0 (0) | 0 |
| 750–1000 m | 223 (5.5) | 0 (0) | 0 |
| 1000–1250 m | 135 (3.3) | 0 (0) | 0 |
| 1250–1500 m | 43 (1.1) | 0 (0) | 0 |
| 1500–1750 m | 12 (0.3) | 0 (0) | 0 |
| 1750–2000 m | 1 (0.0) | 0 (0) | 0 |
| >2000 m | 9 (0.2) | 0 (0) | 0 |

# References

[Dataset] Centers for Disease Control and Prevention (CDC). Social Vulnerability Index. Agency for Toxic Substances and Disease Registry, Geospatial Research, Analysis, and Services Program. 2018. Available from: https://www.atsdr.cdc.gov/placeandhealth/svi/index.

[Dataset] Dewitz J, & U.S. Geological Survey. National Land Cover Database (NLCD) 2019 Products (ver. 2.0, June 2021). U.S. Geological Survey data release. 2021. https://doi.org/10.5066/P9KZCM54.

Federal Emergency Management Agency (FEMA). National Flood Hazard Layer (NFHL). 2021. Available from: https://www.fema.gov/flood-maps/national-flood-hazard-layer.

[Dataset] Hauer ME. Population projections for U.S. counties by age, sex, and race controlled to shared socioeconomic pathway. Scientific Data. 2019. https://doi.org/10.1038/sdata.2019.5.

[Dataset] Homer CG, et al. Conterminous United States land cover change patterns 2001–2016 from the 2016 National Land Cover Database. ISPRS Journal of Photogrammetry and Remote Sensing, 2020;162, 184–199. https://doi.org/10.1016/j.isprsjprs.2020.02.019.

[Dataset] The National Vital Statistics System (NVSS). U.S. Census Populations with Bridged Race Categories. 2021. Available from: https://seer.cancer.gov/popdata/download.html.

[Dataset] U.S. Census Bureau. TIGER/Line Files and Shapefiles. 2019. Available from: https://www.census.gov/geographies/mapping-files.html.

[Dataset] U.S. Department of Agriculture (USDA) Natural Resources Conservation Service (NRCS). National Commodity Crop Productivity Index. 2021. Available from: https://ncsu.maps.arcgis.com/home/item.html?id=9ce0371b69564139b6d13264d2d46a31.

[Dataset] U.S. Geological Survey (USGS). 1 Arc-second Digital Elevation Models (DEMs). USGS National Map 3DEP Downloadable Data Collection. 2017. Available from: https://www.usgs.gov/3d-elevation-program.
